# *Plasmodium* sporozoites require the protein B9 to invade hepatocytes

**DOI:** 10.1016/j.isci.2023.106056

**Published:** 2023-01-25

**Authors:** Priyanka Fernandes, Manon Loubens, Carine Marinach, Romain Coppée, Ludivine Baron, Morgane Grand, Thanh-Phuc Andre, Soumia Hamada, Anne-Claire Langlois, Sylvie Briquet, Philippe Bun, Olivier Silvie

**Affiliations:** 1 Sorbonne Université, INSERM, CNRS, Centre d’Immunologie et des Maladies Infectieuses, CIMI-Paris, Paris, France; 2 Université de Paris, UMR 261 MERIT, IRD, 75006 Paris, France; 3 Sorbonne Université, INSERM, UMS PASS, Plateforme Post-génomique de la Pitié Salpêtrière (P3S), 75013 Paris, France; 4 INSERM U1266, NeurImag Facility, Institute of Psychiatry and Neurosciences of Paris, Paris, France

**Keywords:** Microbiology, Microbiology parasite, Molecular microbiology

## Abstract

*Plasmodium* sporozoites are transmitted to a mammalian host during blood feeding by an infected mosquito and invade hepatocytes for initial replication of the parasite into thousands of erythrocyte-invasive merozoites. Here we report that the B9 protein, a member of the 6-cysteine domain protein family, is secreted from sporozoite micronemes and is required for productive invasion of hepatocytes. The N-terminus of B9 forms a beta-propeller domain structurally related to CyRPA, a cysteine-rich protein forming an essential invasion complex in *Plasmodium falciparum* merozoites. The beta-propeller domain of B9 is essential for sporozoite infectivity and interacts with the 6-cysteine proteins P36 and P52 in a heterologous expression system. Our results suggest that, despite using distinct sets of parasite and host entry factors, *Plasmodium* sporozoites and merozoites may share common structural modules to assemble protein complexes for invasion of host cells.

## Highlights

• Infectious *Plasmodium* sporozoites express the 6-cys protein B9
• B9 is secreted from a specific subset of micronemes
• B9 is required for sporozoite productive invasion of hepatocytes
• B9 contains an essential CyRPA-like beta-propeller domain

## Graphical abstract

**Figure:**
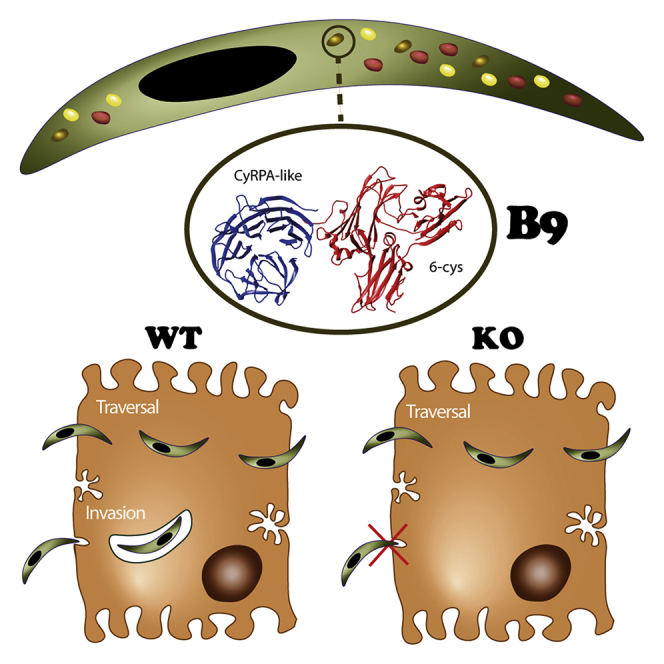

## Introduction

Malaria is caused by *Plasmodium* spp. parasites and still remains a major health and socio-economic problem in endemic countries.^1^ Sporozoites, the mosquito-transmitted forms of the malaria parasite, first infect the liver for an initial and obligatory round of replication, before initiating the symptomatic blood stages. Infection of the liver is clinically silent and constitutes an ideal target for a malaria vaccine. Until now, only a single antigen, the circumsporozoite protein (CSP), had been considered for clinical vaccine development against the extracellular sporozoite stage, with limited success.^2^ Other sporozoite antigens, especially parasite proteins involved in host-parasite interactions, could be considered as potential vaccine targets to prevent sporozoite entry into hepatocytes. This highlights the need to better characterize the molecular mechanisms of sporozoite infection in order to identify new vaccine targets.

Like other Apicomplexan parasites, *Plasmodium* invades host cells using a unique mechanism that involves the sequential secretion of apical organelles, called micronemes and rhoptries, and the formation of a moving junction (MJ) through which the parasite actively glides to enter the cell and form a specialized parasitophorous vacuole (PV) where it further replicates.^3^ Proteins released from micronemes onto the parasite surface are prime candidates to interact with host cell surface receptors, triggering subsequent secretion of the rhoptry content, formation of the MJ, and commitment to productive invasion. However, until now the ligand-receptor interactions mediating *Plasmodium* sporozoite invasion and the nature of the sporozoite MJ have remained enigmatic.^4^

We previously characterized host entry pathways used by human (*P. falciparum*, *P. vivax*) and rodent (*P. yoelii*, *P. berghei*) parasites to infect hepatocytes^5^^,^^6^ and showed that CD81 and the Scavenger Receptor class B type I (SR-BI) define independent entry routes for *P. falciparum* and *P. vivax* sporozoites, respectively.^6^ Remarkably, this alternative usage of host cell receptors is also observed with rodent malaria model parasites, providing robust and tractable experimental systems.^6^^,^^7^ Indeed, *P. yoelii* sporozoites, like *P. falciparum*, strictly require CD81 to infect liver cells, whereas *P. berghei* can alternatively use CD81 or SR-BI for productive invasion.^6^ Only two parasite proteins, P36 and P52, have been identified as being specifically required for productive invasion of hepatocytes.^6^^,^^8^^,^^9^^,^^10^^,^^11^ Using interspecies genetic complementation in mutant *P. berghei* and *P. yoelii* lines, we showed that P36 is a key determinant of host cell receptor usage, establishing for the first time a functional link between sporozoite and host cell entry factors.^6^ The molecular function of P36 remains unknown. One study proposed that P36 interacts with the ephrin receptor EphA2 on hepatocytes to mediate infection,^12^ but direct evidence for such an interaction is lacking, and EphA2 was later shown to be dispensable for sporozoite productive invasion.^13^ Interestingly, interspecies genetic complementation experiments showed that *P. berghei* Δ*p52*Δ*p36* mutants complemented with PyP52 and PyP36 exhibit a *P. yoelii*-like phenotype as they preferentially infect CD81-expressing cells.^6^ However, while *P. yoelii* sporozoites are unable to infect hepatocytes in the absence of CD81, complemented *P. berghei* mutants retain a residual invasion capacity in CD81-deficient cells.^6^ Furthermore, genetic complementation with *P. falciparum* or *P. vivax* P52 and P36 does not restore infectivity of Δ*p52*Δ*p36 P. berghei* sporozoites.^6^ These results strongly suggest that additional parasite factors contribute to receptor-dependent productive invasion.

P36 and P52 both belong to the so-called 6-cysteine domain protein family, which is characterized by the presence of one or several 6-cysteine (6-cys) domains.^14^ 6-cys domains are ∼120 amino acid-long domains containing four or six conserved cysteine residues that respectively form two or three disulphide bonds resulting in a beta-sandwich fold.^14^ *Plasmodium* spp. possess 14 members of the 6-cys protein family.^15^ *Plasmodium* 6-cys proteins are typically expressed in a stage-specific manner and have been implicated in protein-protein interactions in *P. falciparum* merozoites,^16^^,^^17^ gametocytes,^18^^,^^19^ ookinetes,^20^ and sporozoites.^11^ Proteomic studies have shown that, in addition to P36 and P52, *Plasmodium* sporozoites express three other 6-cys proteins, P12p, P38, and B9.^21^^,^^22^^,^^23^^,^^24^ While the contribution of P12p and P38 had not been studied until now, a previous study reported that the protein B9 is not expressed in sporozoites due to translational repression and is not required for sporozoite invasion of hepatocytes but is needed for early maintenance of the PV.^15^

Here, we systematically analyzed the role of P12p, P38, and B9 during sporozoite invasion, using a reverse genetics approach based on our gene out marker out (GOMO) strategy.^25^ We report that *b9* gene deletion totally abrogates sporozoite infectivity, while *p12p* and *p38* are dispensable for hepatocyte infection in both *P. berghei* and *P. yoelii*. We show that B9 is a sporozoite micronemal protein and that B9-deficient sporozoites fail to productively invade hepatocytes. Secondary structure analysis and protein structure modeling indicate that B9 is a hybrid protein containing a CyRPA-like beta-propeller domain in addition to noncanonical 6-cys domains. Structure-guided mutagenesis reveals that the propeller domain is not associated with host cell receptor usage but is essential for sporozoite infectivity, being required for adequate protein expression and/or function, possibly through the assembly of supramolecular protein complexes with the 6-Cys proteins P36 and P52.

## Results

### Analysis of the repertoire of *Plasmodium* sporozoite 6-cys proteins suggests that P36, P52, and B9 are employed by infectious sporozoites only

In order to define the repertoire of 6-cys proteins expressed at the sporozoite stage, we first analyzed the proteome datasets of *P. falciparum,*^22^^,^^23^ *P. vivax*,^24^ *P. yoelii*,^23^ and *P. berghei*^21^ sporozoites. As expected, P36 and P52 were identified by mass spectrometry in sporozoites from all four species. Interestingly, three other 6-cys proteins, P12p, P38, and B9, were consistently identified across the datasets. Among this core of five 6-cys proteins, P12p and P38 have been identified in the surface proteome of *P. falciparum* sporozoites, with P12p being quantitatively enriched on the surface of activated parasites in the presence of bovine serum albumin.^26^ Interestingly, P12p and P38 do not seem to be uniquely employed by sporozoites as they have been detected in *P. falciparum* asexual and sexual blood stages^27^^,^^28^^,^^29^^,^^30^^,^^31^ and in *P. berghei* gametocytes,^32^ respectively. In contrast, P36, P52, and B9 were only identified in sporozoites, and a recent study identified P36, P52, and B9 as upregulated in infectious sporozoites (UIS) proteins in *P. falciparum* and *P. yoelii*, while P12p and P38 were also detected in oocyst-derived sporozoites.^33^ These observations suggest that B9, like P36 and P52, may play a role in mature sporozoites.

### Reverse genetics analysis in rodent malaria parasites shows that *b9* (but not *p12p* and *p38*) is essential for sporozoite infectivity

**Figure 1:**
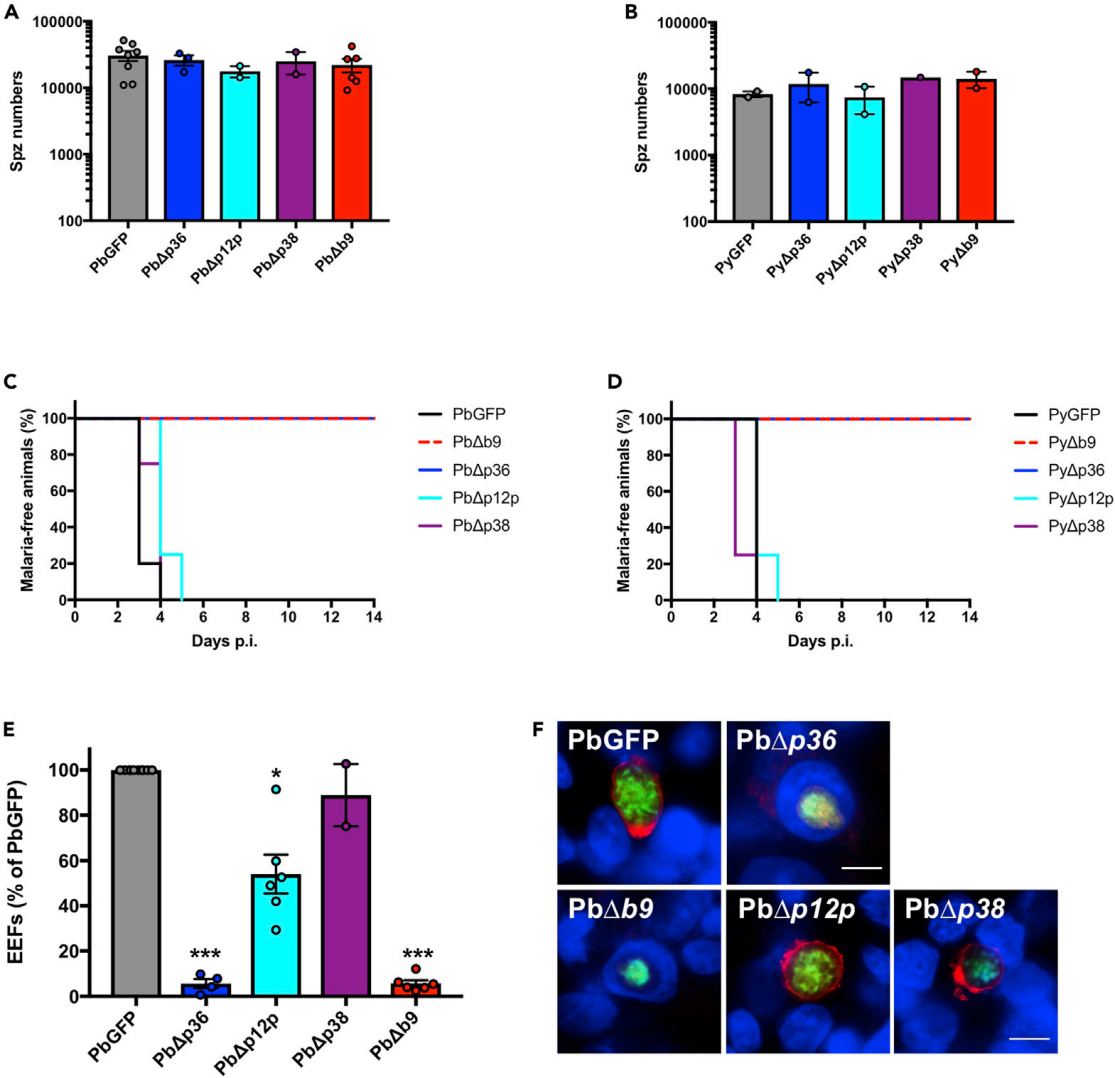
Deletion of *b9* but not *p12p* or *p38* genes abrogates sporozoite infectivity in *P. berghei* and *P. yoelii* (A) Number of sporozoites isolated from the salivary glands of mosquitoes infected with PbGFP, PbΔ*p36*, PbΔ*p12p,* PbΔ*p38*, or PbΔ*b9* parasites (mean ± SEM, in log scale; p = 0.67, one-way ANOVA). Each dot represents the mean number of sporozoites per female mosquito in one experiment. (B) Number of sporozoites isolated from the salivary glands of mosquitoes infected with PyGFP, PyΔ*p36*, PyΔ*p12p*, PyΔ*p38*, or PyΔ*b9* parasites (mean ± SEM, in log scale; p = 0.66, one-way ANOVA). Each dot represents the mean number of sporozoites per female mosquito in one experiment. (C) Kaplan-Meier analysis of time to patency in C57BL/6 mice (n = 5) after intravenous injection of 10^4^ PbGFP, PbΔ*p36*, PbΔ*p12p,* PbΔ*p38*, or PbΔ*b9* sporozoites. Mice were followed daily for the appearance of blood-stage parasites (p = 0.0001, Log rank Mantel-Cox test). (D) Kaplan-Meier analysis of time to patency in BALB/c mice (n = 5) after intravenous injection of 10^4^ PyGFP, PyΔ*p36*, PyΔ*p12p*, PyΔ*p38*, or PyΔ*b9* sporozoites. Mice were followed daily for the appearance of blood-stage parasites (p < 0.0001, Log rank Mantel-Cox test). (E) Infection rates were determined by quantification of EEFs (GFP-positive cells) 24 h after infection of HepG2 cell cultures with PbGFP, PbΔ*p36*, PbΔ*p12p,* PbΔ*p38*, or PbΔ*b9* sporozoites. Data are represented as % of PbGFP control (mean ± SEM). Each dot represents the mean value in one experiment. ∗∗∗p < 0.001 as compared to PbGFP (one-way ANOVA followed by Dunnett’s multiple comparisons test). (F) Immunofluorescence images of HepG2 cells infected with PbGFP, PbΔ*p36*, PbΔ*p12p,* PbΔ*p38*, or PbΔ*b9* parasites expressing GFP (green) and labeled with anti-UIS4 antibodies (red) and Hoechst 33342 (blue), 48 h postinfection. PbGFP, PbΔ*p12p*, and PbΔ*p38* are surrounded by a UIS4-positive PV membrane (red), while PbΔ*p36* and PbΔ*b9* parasites are intranuclear and lack a UIS4-positive PVM. Scale bar, 10 μm. See also Figures S1 and S2, and Table S6 for quantitative source data.

A previous study reported that B9 is not expressed in sporozoites and is required for early liver-stage development but not host cell invasion.^15^ The contribution of P12p and P38 during sporozoite invasion has not been investigated so far, although the *p38* gene could be deleted in *P. berghei* without any detectable phenotypic defect during blood-stage parasite growth and transmission to mosquitoes.^34^^,^^35^ Given the consistent detection of P12p, P38, and B9 proteins in sporozoites by mass spectrometry, we sought to determine the functional importance of these proteins in *P. berghei* and *P. yoelii* sporozoites using a reverse genetics approach. We used our GOMO strategy^25^ to replace genes of interest, through homologous recombination, with a GFP expression cassette under the control of a constitutive HSP70 promoter, to facilitate monitoring of host cell invasion (Figure S1A). Targeting vectors were assembled by inserting 5’ and 3’ homology fragments of *P. berghei* or *P. yoelii p12p* (PBANKA_0111100; PY17X_0112700), *p38* (PBANKA_1107600; PY17X_1108700), and *b9* (PBANKA_0808100; PY17X_0811300) genes in the GOMO-GFP plasmid^25^ and used to transfect wild-type (WT) *P. berghei* (ANKA) or *P. yoelii* (17XNL) blood-stage parasites. We then applied the GOMO selection strategy, consisting of positive selection with pyrimethamine, negative selection with 5-fluorocytosine, and flow cytometry-assisted parasite sorting, as previously described.^25^ Pure populations of GFP-expressing drug-selectable marker-free PbΔ*p12p*, PbΔ*p38*, PbΔ*b9*, PyΔ*p12p*, PyΔ*p38*, and PyΔ*b9* parasite lines were obtained, confirming that none of the targeted genes are essential during blood-stage replication of the parasite. Genotyping by PCR confirmed gene deletion and excision of the drug-selectable marker cassette, as desired, in all parasite lines (Figures S1B–S1H). All the mutants could be transmitted to mosquitoes and produced normal numbers of salivary gland sporozoites, similar to Δ*p36* parasites (Figures 1A and 1B). We then assessed the infectivity of the *P. berghei* and *P. yoelii* mutant lines in C57BL/6 and BALB/c mice, respectively. C57BL/6 mice injected with 10,000 PbΔ*p12p* or PbΔ*p38* sporozoites all developed a patent blood-stage infection, like the parental PbGFP parasites (Figure 1C). Similarly, BALB/c mice injected with 10,000 PyΔ*p12p* or PyΔ*p38* sporozoites all developed a patent blood-stage infection (Figure 1D). In sharp contrast, none of the animals injected with *P. berghei* or *P. yoelii* Δ*b9* sporozoites developed parasitemia, phenocopying the Δ*p36* mutants (Figures 1C and 1D). Abrogation of Δ*b9* sporozoite infectivity was also observed *in vitro* in hepatocyte cell lines. Fluorescence-activated cell sorting (FACS) analysis 24 hours postinfection revealed a dramatic reduction in the number of PbΔ*b9* exoerythrocytic forms (EEFs) in comparison to control PbGFP or PbΔ*p12p* and PbΔ*p38* sporozoites in HepG2 cells, which was similar to the reduction observed with PbΔ*p36* mutants (Figure 1E). Using antibodies specific for UIS4, a marker of the PV membrane (PVM) that specifically labels productive vacuoles,^3^^,^^36^ we confirmed that, in contrast to Δ*p12p* and Δ*p38* mutants*,* Δ*b9* parasites were not able to form productive vacuoles (Figures 1F and S2). Together, these results show that *b9* is essential for sporozoite infection of the liver both *in vivo* and *in vitro*, corroborating the results of a previous study,^15^ and that *p12p* and *p38* genes on the contrary are dispensable for parasite invasion and liver-stage development.

### B9 is required for sporozoite invasion

**Figure 2:**
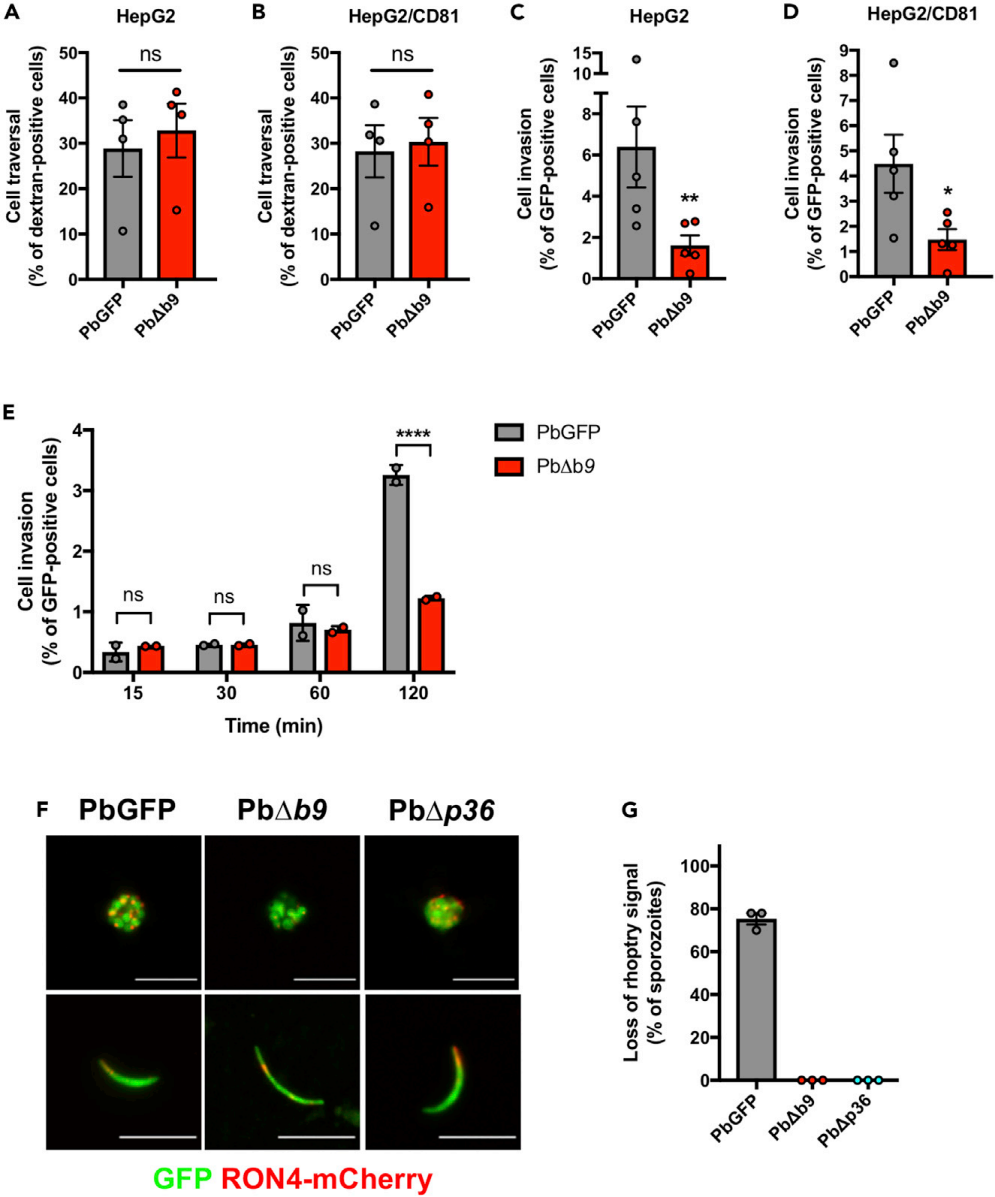
Sporozoites require B9 for productive invasion of host cells (A and B) Sporozoite cell traversal activity was analyzed in HepG2 (A) and HepG2/CD81 (B) cell cultures incubated for 3 h with PbGFP or PbΔ*b9* sporozoites in the presence of rhodamine-labeled dextran. The number of traversed (dextran-positive) cells was determined by FACS. The data are represented as % of dextran-positive cells (mean ± SEM). Each dot represents the mean value in one experiment. ns, nonsignificant (two-tailed ratio paired t test). (C and D) Sporozoite invasion rates were determined in HepG2 (C) and HepG2/CD81 (D) cell cultures incubated for 3 h with PbGFP or PbΔ*b9* sporozoites. The total percentage of invaded (GFP-positive) cells, encompassing both cell traversal and productive invasion, was determined by FACS. The data are represented as % of GFP-positive cells (mean ± SEM). Each dot represents the mean value in one experiment. ∗p < 0.05, ∗∗p < 0.01 (two-tailed ratio paired t test). (E) Sporozoite invasion rates were determined in HepG2 cell cultures incubated for 15 to 120 min with PbGFP or PbΔ*b9* sporozoites. The data are represented as % of GFP-positive cells (mean ± SEM). Each dot represents the mean value in one experiment. Ns, nonsignificant; ∗∗∗∗p < 0.0001 (two-way ANOVA). (F) Fluorescence microscopy images of RON4-mCherry-expressing PbGFP, PbΔ*b9*, and PbΔ*p36* erythrocytic schizonts (upper panels) and salivary gland sporozoites (lower panels), showing direct detection of GFP (green) and mCherry (red). Scale bar, 10 μm. (G) Depletion of rhoptry proteins was assessed by fluorescence microscopy examination of HepG2 cells incubated for 3 h with RON4-mCherry-expressing PbGFP, PbΔ*b9*, or PbΔ*p36* sporozoites. Results are expressed as the percentage of parasites without detectable RON4-mCherry signal. See also Table S6 for quantitative source data.

After infection of HepG2 cell cultures with Δ*b9* sporozoites, only very low numbers of intracellular parasites were observed, all of which were seemingly intranuclear and lacked a UIS4-labeled PVM, similar to the Δ*p36* mutants (Figures 1F and S2). Our results contrast with previous reports where mutant EEFs devoid of a PVM were observed in the cytoplasm of infected cells.^15^^,^^37^ This discrepancy is likely due to differences in the hepatoma cell lines that were used (Huh-7 versus HepG2). Intranuclear EEFs in HepG2 cells are known to result from cell traversal events.^38^ Accordingly, a cell wound-repair assay confirmed that the cell traversal activity of Δ*b9* sporozoites is not different to PbGFP parasites, in both HepG2 and HepG2/CD81 cells (Figures 2A and 2B). In contrast, direct quantification of invaded cells by FACS at 3 h postinfection revealed that host cell invasion by Δ*b9* sporozoites is greatly impaired in both cell types (Figures 2C and 2D). Further examination of the invasion kinetics revealed low invasion rates with both PbGFP and Δ*b9* sporozoites in the early time points (15–60 min) (Figure 2E), when sporozoites are in the traversal mode.^3^^,^^6^ At the 2 hour time point, the percentage of PbGFP-invaded cells was markedly increased (Figure 2E), reflecting commitment to productive invasion and accumulation of sporozoites inside PV.^3^^,^^6^ A similar increase was not observed with Δ*b9* parasites (Figure 2E)*,* suggesting a defect in productive invasion, similar to P52/P36-deficient sporozoites.^6^ Productive host cell invasion is associated with discharge of the sporozoite rhoptries, resulting in depletion of the rhoptry proteins RON2 and RON4.^3^^,^^39^ To visualize the rhoptries in B9-deficient parasites, we genetically modified the *ron4* locus in the PbΔ*b9* mutant line to replace the endogenous RON4 by a RON4-mCherry fusion by double homologous recombination (Figures S3A and S3D). In parallel, we also genetically modified parental PbGFP and mutant PbΔ*p36* parasites, using the same RON4-targeting vector (Figures S3B and S3C). Examination of PbGFP/*RON4-mCherry*, PbΔ*b9/RON4-mCherry*, and PbΔ*p36/RON4-mCherry* by fluorescence microscopy confirmed expression of the rhoptry marker in merozoites and sporozoites, as expected^39^ (Figure 2F). We then performed invasion assays in HepG2 cells and analyzed the presence of the RON4-mCherry rhoptry marker by fluorescence microscopy. As expected, the RON4-mCherry signal was lost in a vast majority of intracellular PbGFP/*RON4-mCherry* sporozoites as a result of rhoptry discharge during productive invasion (Figure 2G). In sharp contrast, RON4-mCherry was detected in all examined PbΔ*b9* and PbΔ*p36* intracellular sporozoites, indicating that sporozoites lacking B9 or P36 invade cells without secreting their rhoptries, i.e. through traversal mode only. Altogether, these data demonstrate that genetic deletion of B9 abrogates productive host cell invasion by sporozoites, phenocopying the lack of P36. Our data also show that B9, like P36, is essential for both CD81-dependent and CD81-independent sporozoite entry.

### B9 is secreted from the sporozoite micronemes

The phenotype of Δ*b9* mutants, combined with proteomic data, implies that the protein B9 is expressed in *P. berghei* sporozoites and plays a crucial role during host cell productive invasion, unlike previously thought.^15^ In order to confirm the expression of B9 at the protein level and define its localization, we genetically modified the endogenous *b9* locus in *P. berghei* (PbGFP) to insert a triple Flag epitope in the protein-coding sequence, through double homologous recombination (Figure S4A). Because B9 is predicted to be glycosylphosphatidylinositol (GPI) anchored, we inserted the 3xFlag tag towards the C-terminus of the protein, downstream of the putative 6-cys domains but upstream of the predicted omega site (aspartate residue at position 826). Correct integration of the construct was confirmed by PCR on genomic DNA from B9-Flag blood-stage parasites (Figure S4B). Importantly, we observed no defect in sporozoite development (Figure S4C) and infectivity (Figure S4D) in the B9-Flag line, demonstrating that the insertion of a 3xFlag epitope in B9 sequence had no detrimental effect on the protein function.

**Figure 3:**
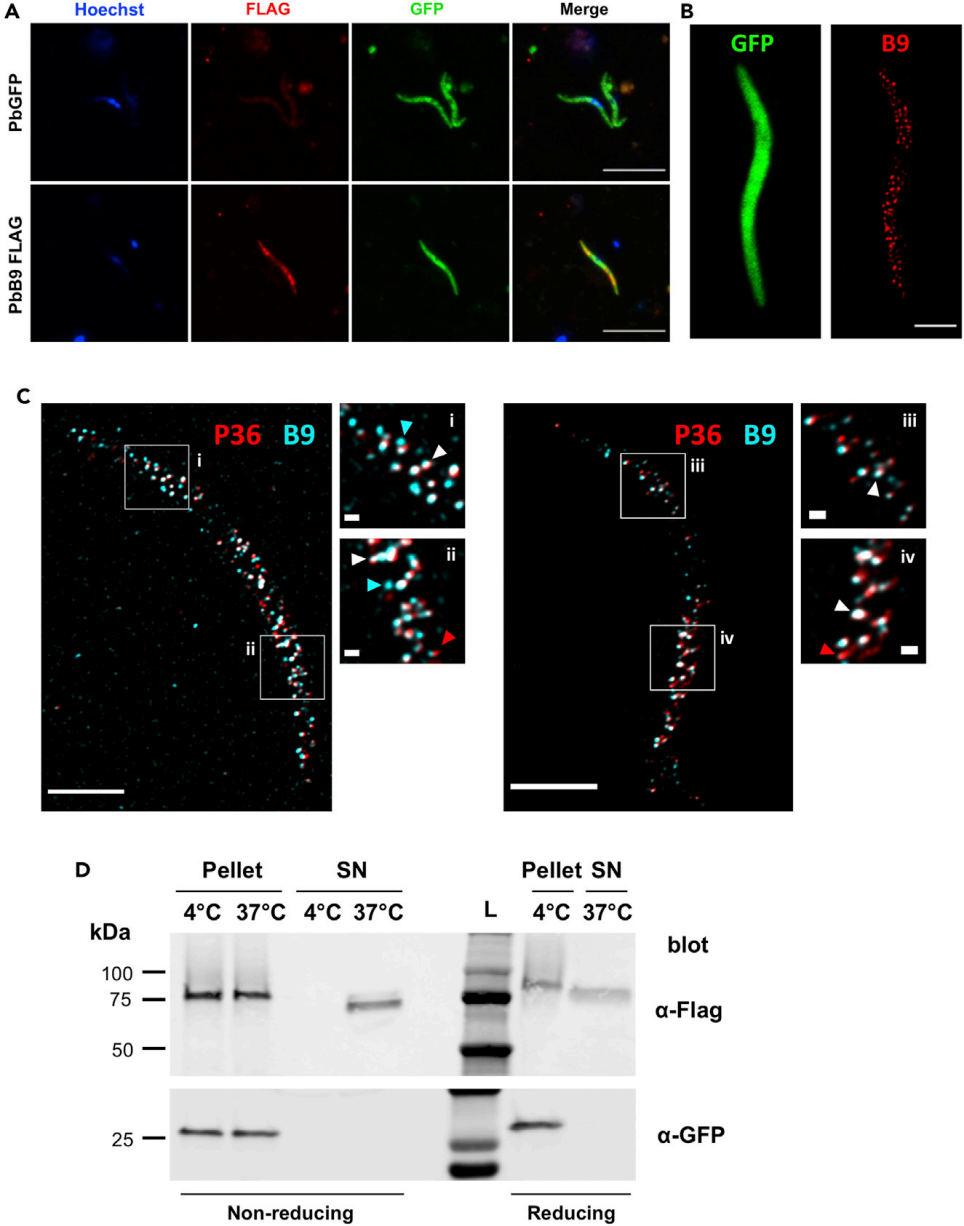
B9 localizes to a subset of sporozoite micronemes and is secreted upon parasite activation (A) Immunofluorescence analysis of PbGFP and PbB9-Flag sporozoites labeled with anti-Flag antibodies (red). Parasites express GFP (direct detection, green) and nuclei were stained with Hoechst 33342 (blue). Scale bar, 10 μm. (B) Localization of B9 in sporozoites. First panel, confocal image of GFP (direct detection, green); second panel, visualization of B9-Flag (with anti-Flag, red) using 2D STED (maximum intensity projection). Scale bar, 2 μm. (C) STED images of sporozoites expressing B9-Flag and P36-mCherry, labeled with anti-mCherry (red) and anti-Flag (cyan) antibodies. Scale bars, 2 μm (200 nm in insets). Some micronemes are labeled for both B9 and P36 (white arrowheads). Others are stained only for B9 (cyan arrowheads) or P36 (red arrowheads). (D) Immunoblot of B9-Flag sporozoite pellets and supernatants in control conditions (4°C) or after stimulation of microneme secretion (37°C), using anti-Flag or anti-GFP antibodies. The data shown are representative of three independent experiments. See also Figure S5.

Immunofluorescence with anti-Flag antibodies revealed that B9 is readily detected in B9-Flag salivary gland sporozoites, with a distribution pattern typical of a micronemal protein (Figure 3A). As a control, parental PbGFP sporozoites showed no signal with the Flag antibody, confirming the specificity of the labeling (Figure 3A). Super-resolution microscopy using stimulated emission depletion (STED) showed that B9 distributes in numerous vesicles localized on each side of the nucleus, consistent with B9 being a micronemal protein (Figure 3B). Interestingly, B9 colocalized in part with P36 (Figures 3C and S5A) but not with the thrombospondin-related anonymous protein (TRAP) (Figure S5B) or the apical membrane antigen 1 (AMA1) (Figure S5C), suggesting that B9 is present in a specific subset of micronemes in salivary gland sporozoites. We next analyzed the fate of B9 upon activation of sporozoite microneme secretion, by western blot. In nonactivated control parasites, B9 was detected as a single band between 75 and 100kDa, in both reducing and nonreducing conditions, consistent with the expected size of the protein (∼95 kDa) (Figure 3D). Upon stimulation of microneme secretion, B9 was also recovered in the supernatant fraction as a slightly smaller band, indicating that B9 is secreted from sporozoites upon activation, possibly after enzymatic processing (Figure 3D). We failed to detect B9 on the surface of B9-Flag sporozoites by immunofluorescence, irrespective of parasite activation, suggesting that following microneme secretion, B9 is mainly released as a shed protein.

### B9 contains a CyRPA-like beta-propeller domain

**Figure 4:**
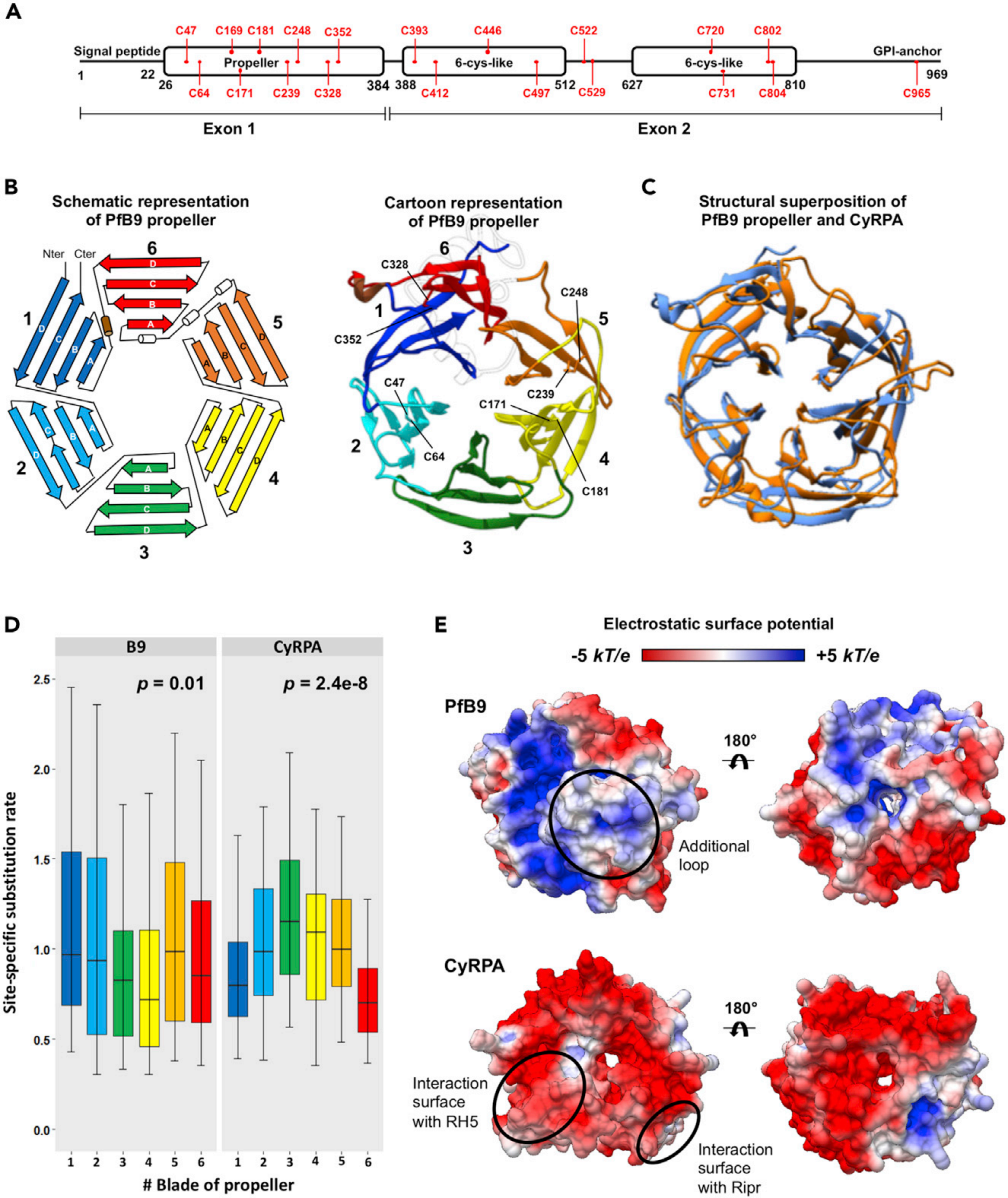
Structural and evolutionary features of B9 propeller (A) Predicted B9 conserved domains. PfB9 was used as the reference sequence. Cysteines are indicated in red. The delimitation of the domains is based on the HHpred results. B9 is composed of two exons, the first one covering the whole propeller domain. (B) Predicted tertiary structure of PfB9 propeller. The predicted model is indicated as a schematic representation (*left*) and as a cartoon (*right*). Each of the six blades is indicated with a specific color, labeled 1 to 6, and is composed of four-stranded anti-parallel beta-sheet, labeled A to D. The four disulfide bridges found in PfB9 are indicated. The long loop connecting blades 5 and 6 in the cartoon representation is transparent for ease of representation. (C) Structural superposition of PfB9 propeller with CyRPA. PfB9 and CyRPA are respectively colored in blue and orange. Both superposition and RMSD calculation were based on all Cɑ atoms using the *MatchMaker* function in UCSF Chimera. (D) Conservation level of the six blades of B9 propeller and CyRPA. Site-specific rates were estimated using the GP4Rate tool and were compared between the six blades using nonparametric Kruskal-Wallis *H* test. Box boundaries represent the first and third quartiles, and the length of whiskers corresponds to 1.5 times the interquartile range. (E) Electrostatic surface potential of PfB9 propeller and CyRPA structures, estimated with the APBS method. Electrostatic potential values are in units of kT/e at 298 K, on a scale of −5 kT/e (red) to +5 kT/e (blue). White color indicates a neutral potential. The missing charges were added using the Add Charge function implemented in USCF Chimera. The additional long loop connecting blades 5 and 6 of PfB9 propeller and the interaction surfaces of CyRPA with Rh5 and Ripr are indicated with circles. See also Figures S6–S9.

To get more insights into B9 properties, we investigated sequence and structural features of the protein using *P. falciparum* B9 as the reference sequence. Both hydrophobic cluster analysis and secondary structure prediction of B9 suggested that the whole sequence contains some strand and helix structures (Figures S6A and S6B). However, no annotated conserved domain was detected at the sequence level using InterPro. In sharp contrast, three domains were predicted at the structural level using HHpred: a N-terminus propeller domain similar to that of CyRPA (e-value: 5.4e-03) encoded by the first exon and two putative but poorly supported 6-cys domains encoded by the second exon (e-value > 1) (Figure 4A). CyRPA is a cysteine-rich protein expressed in *P. falciparum* merozoites, where it forms a protein complex that is essential for invasion of erythrocytes.^40^^,^^41^ B9 is enriched in cysteines, nine being located in the predicted propeller domain that we suppose are involved in the formation of disulphide bonds in a similar manner to CyRPA,^42^ to stabilize the protein structure (Figure 4A).

To explore the structural features of the B9 propeller, we predicted the tertiary structure of PfB9 propeller (covering positions 26 to 386) by homology modeling using CyRPA as a template structure^42^ (PDB ID: 5TIH; Figure S7). As expected, PfB9 adopted a six-bladed propeller structure, with each blade being composed of four-stranded antiparallel beta-sheets (Figure 4B). Four disulphide bonds were predicted within the blades which may stabilize each individual blade of the PfB9 propeller (C47-C64, C171-C181, C239-C248, and C328-C352; Figure 4B). Furthermore, a long loop connecting blades 5 and 6 and containing three putative short helices was observed in the PfB9 propeller, which was not found in CyRPA and in most *Plasmodium* B9 proteins (such as PbB9 and PyB9; Figure S8). This partially structured region is supported by intrinsic disorder prediction (Figure S6C), in line with another characteristic of CyRPA, where the loop located on blade 5 likely becomes disordered to accommodate occupancy by a helix of Rh5.^41^ The model superimposed well with the CyRPA structure, except for some blade- and strand-connecting loops (root-mean-square deviation [RMSD]: 3.8 Å; Figure 4C). This similar fold, in addition to the binding activities of CyRPA (targeting Rh5 and Ripr^41^), suggests that the B9 propeller may promote protein-protein interactions.

Because CyRPA is functionally annotated and its binding properties are known, we checked whether the B9 propeller and CyRPA shared a common evolutionary history, which could help to predict the functional sites in the B9 propeller. For this, we generated two datasets consisting of distinct *Plasmodium* B9 (n = 23) or CyRPA (n = 18) sequences (Table S1). Multiple sequence alignments and corresponding phylogenetic trees of these datasets (Figure S9) were then used concomitantly with their respective tertiary structures to estimate spatially correlated site-specific substitution rates using the GP4Rate tool (Table S2). The six blades were found to be heterogeneously conserved over time for both B9 and CyRPA (Kruskal-Wallis *H* test: B9: *p* = 0.01; CyRPA: *p* = 2.4e-8; Figure 4D). Interestingly, we noticed distinct patterns of evolution between the two proteins: the most conserved blades of B9 propeller (3 and 4) are the less conserved ones in CyRPA (Figure 4D). Because CyRPA interacts with Ripr through its most conserved blade,^41^ i.e. 6 (Figure 4D), we logically hypothesize that the blades 3 and 4 of the B9 propeller may target putative partners. Finally, in concordance with different evolutionary histories, we note that the PfB9 propeller and CyRPA display a dissimilar electrostatic surface potential. While almost the entire surface of CyRPA (including the regions mediating interactions with Rh5 and Ripr) is electronegative, some parts of the PfB9 propeller are electropositive (Figure 4E), thus suggesting different binding properties.

### The propeller domain of B9 is required for sporozoite infectivity

**Figure 5:**
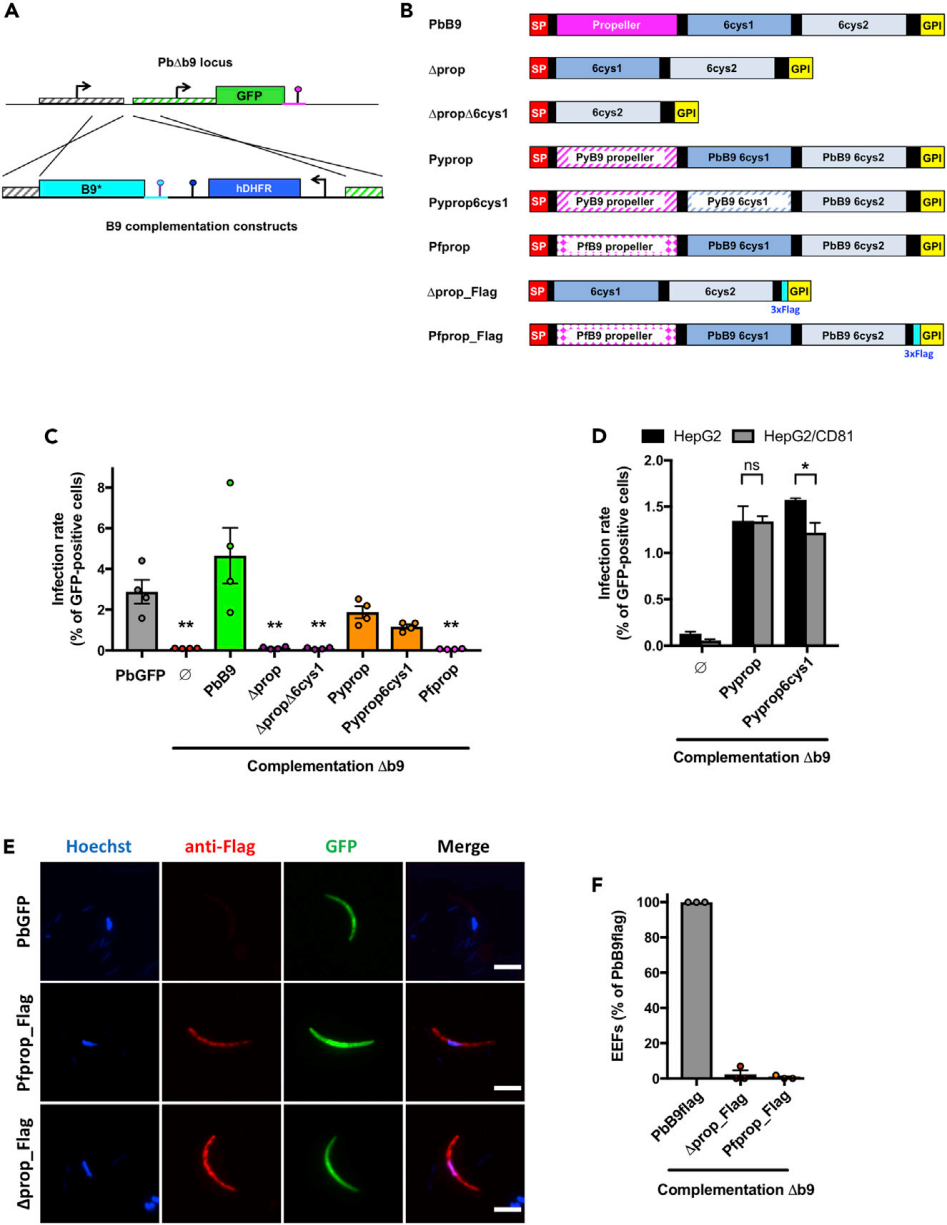
The propeller domain of B9 is required for sporozoite infectivity but does not restrict host receptor usage (A) Strategy used to genetically complement PbΔ*b9* with different versions of B9 (indicated as B9∗) by double crossover homologous recombination. (B) Schematic representation of the B9 constructs used for genetic complementation. SP, signal peptide, GPI, glycosylphosphatidylinositol. (C) Infection rates were determined by quantification of EEFs (GFP-positive cells) 24 h after infection of HepG2 cell cultures with sporozoites of PbGFP, PbΔ*b9*, or PbΔ*b9* complemented with PbB9, Δprop, ΔpropΔ6cys1, PyProp, PyProp6cys1, or PfProp constructs. Data are represented as % of GFP-positive cells (mean ± SEM). Each dot represents the mean value in one experiment. ∗∗p < 0.01 as compared to PbGFP (one-way ANOVA followed by Dunnett’s multiple comparisons test). (D) Infection rates in HepG2 or HepG2/CD81 cells infected with PbΔ*b9* or PbΔ*b9* complemented with PyProp or Pyprop6cys1 constructs were determined 24 h postinfection. The results show the percentage of invaded (GFP-positive) cells as determined by FACS (mean ± SEM). ns, nonsignificant, ∗p < 0.05 (two-way ANOVA followed by Sidak’s multiple comparisons test). (E) Immunofluorescence analysis of sporozoites from PbGFP and PbΔ*b9* complemented with Pfprop-Flag or Δprop-Flag constructs, labeled with anti-Flag antibodies (red). Parasites express GFP (green) and nuclei were stained with Hoechst 33342 (blue). Scale bars, 5 μm. (F) Infection rates were determined 24 h after infection of HepG2 cell cultures with sporozoites of PbB9-Flag or PbΔ*b9* complemented with Δprop-Flag or Pfprop-Flag constructs. Results are expressed as % of control (PbB9-Flag). See also Figures S10 and S11, and Table S6 for quantitative source data.

We next sought to define the functional importance of the predicted propeller domain, using a structure-guided genetic complementation strategy to evaluate the functionality of truncated B9 proteins (Figure 5A). We assembled various constructs encoding the entire or partially deleted B9, all containing an intact signal peptide and C-terminus sequences to ensure correct secretion and GPI anchoring of the protein (Figure 5B). Constructs were used for transfection of the drug-selectable marker-free PbΔ*b9* mutant line. After confirmation of correct integration by genotyping PCR (Figure S10), genetically complemented parasites were transmitted to mosquitoes, and sporozoites were tested for infectivity in cell cultures. Complementation of PbΔ*b9* sporozoites with a construct encoding the entire PbB9 fully restored sporozoite infectivity in HepG2 cell cultures (Figure 5C), validating the genetic complementation approach. In contrast, parasites complemented with a truncated B9 lacking the propeller domain, alone or in combination with the first 6-cys domain, were not infectious, phenocopying the parental B9-deficient parasites (Figure 5C). These results show that the propeller domain is required for sporozoite infectivity. Interestingly, chimeric B9 versions where the propeller domain of PbB9 was replaced by the equivalent sequence from PyB9 (Pyprop, Pyprop6cys1; Figure 5B) restored sporozoite infectivity (Figure 5C). In contrast, substitution of the PfB9 propeller domain for the PbB9 propeller (Pfprop; Figure 5B) did not restore infectivity in complemented parasites (Figure 5C). Complementation with the PyB9 propeller domain restored infection in both HepG2 cells, which express SR-B1 but not CD81, and HepG2/CD81 cells, which express both receptors,^6^ suggesting that the B9 propeller domain does not restrict host cell receptor usage (Figure 5D). To exclude a defect in protein expression with the nonfunctional constructs, we generated two additional parasite lines expressing Flag-tagged version of the Δprop and Pfprop B9 proteins (Figures 5B and S10). The Δprop-Flag and Pfprop-Flag proteins were detected in transgenic sporozoites by immunofluorescence (Figure 5E) and western blot (Figure S11), indicating that truncation of the propeller domain does not totally impair protein expression. However, both proteins were expressed at low levels as compared to PbB9-Flag, as evidenced by western blot (Figure S11). Both Δprop-Flag and Pfprop-Flag constructs failed to restore infectivity in PbΔ*b9* sporozoites (Figure 5F), as observed with untagged proteins. This confirms that the beta-propeller domain is essential for sporozoite infectivity, by enabling adequate protein expression and/or regulating B9 function.

### The propeller domain of B9 interacts with P36 and P52 in a heterologous system

Our structural modeling revealed that B9 contains an N-terminus beta-propeller domain structurally similar to CyRPA. In *P. falciparum* merozoites, CyRPA interacts with Rh5 and Ripr to form a complex that is essential for invasion of erythrocytes.^40^^,^^41^^,^^43^ While Ripr is conserved among *Plasmodium* species, CyRPA is found in primate but not rodent parasites, and Rh5 is restricted to *P. falciparum* and other *Laverania* species.^44^ As Rh5 and Ripr are not expressed by sporozoites,^21^^,^^23^^,^^24^ we hypothesized that B9 might be involved in the formation of distinct protein complexes in sporozoites. To test this hypothesis, we first performed co-immunoprecipitation (coIP) experiments with anti-Flag antibodies, using protein extracts from B9-Flag sporozoites, followed by protein identification by mass spectrometry. PbGFP sporozoites were used as a control. B9 was the only protein consistently identified in five independent biological replicates by mass spectrometry (Table S3). We considered that B9 might interact with other sporozoite proteins only at the time of host cell invasion, similarly to CyRPA, which interacts with Rh5 following secretion of merozoite apical organelles.^40^ To test this hypothesis, we performed coIP experiments on supernatants of HepG2 cell cultures incubated with B9-Flag sporozoites, using uninfected cultures as a control. Again, B9 was the only protein specifically identified in supernatants from infected cultures (Table S3). While these results confirm that B9 is secreted during infection, it is likely that protein amounts released in the culture supernatants are not sufficient to identify interacting proteins by mass spectrometry after immunoprecipitation.

**Figure 6:**
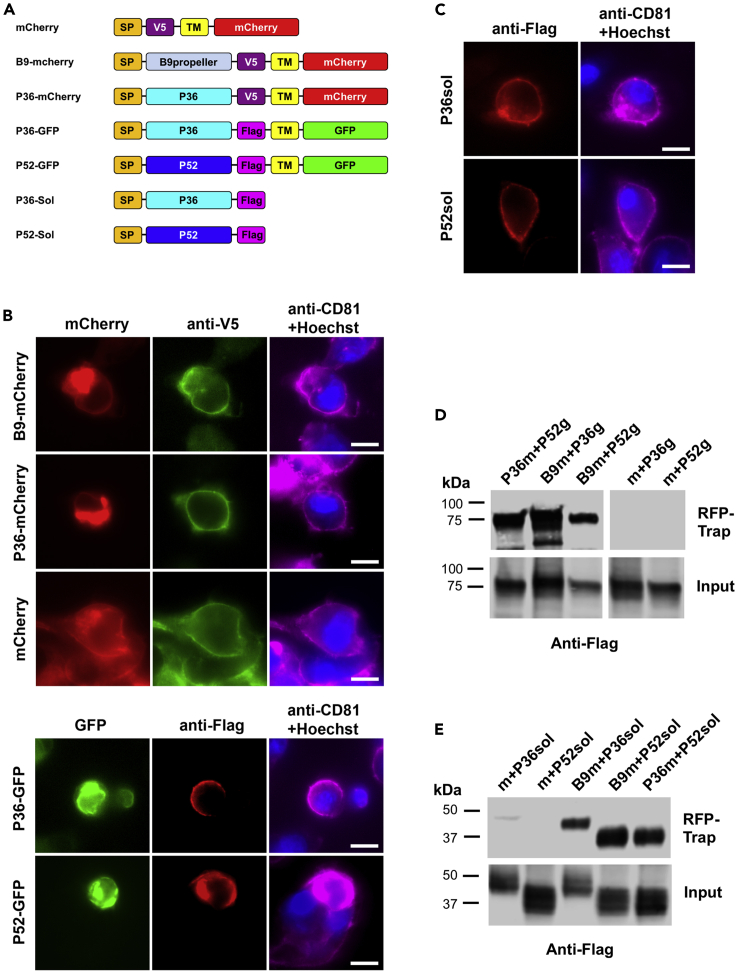
The propeller domain of B9 interacts with P36 and P52 in a heterologous expression system (A) Schematic representation of the constructs used for heterologous expression in mammalian cells. SP, signal peptide from the bee venom melittin; TM, transmembrane domain and C-terminal portion of mouse Glycophorin A. (B) Hepa1-6 cells were examined by fluorescence microscopy 24 h following transfection with mCherry- and GFP-tagged constructs. Cells were fixed without permeabilization and labeled with anti-CD81, anti-V5 or anti-Flag antibodies, and Hoechst 33342. Scale bars, 10 μm. (C) Hepa1-6 cells transfected with P36-Sol and P52-Sol constructs were fixed without permeabilization and labeled with anti-CD81, anti-Flag antibodies, and Hoechst 33342. Scale bars, 10 μm. (D and E) Hepa1-6 cells were transiently transfected with constructs encoding mCherry (m), B9-mCherry (B9m), or P36-mCherry (P36m) constructs, together with P36-GFP (P36g), P52-GFP (P52g), P36sol, or P52sol constructs. Following immunoprecipitation of mCherry-tagged proteins, co-immunoprecipitated proteins (RFP-trap) and total extracts (input) were analyzed by western blot using anti-Flag antibodies. The data shown are representative of three independent experiments.

Therefore, we opted for an alternative strategy based on heterologous expression of sporozoite proteins in mammalian cells, to test for potential interactions between B9 and the 6-cys proteins P36 and P52 as candidate partners, a choice motivated by the shared phenotype of gene-deletion mutants. For this purpose, we used a surface display approach to express *P. berghei* proteins on the surface of Hepa1-6 cells after transient transfection.^45^ Codon-optimized versions of the propeller domain of PbB9 (amino acids 31-348) or the tandem 6-cys domains of PbP36 (amino acids 67-352) were fused at the N-terminus to the signal peptide of the bee venom melittin (BVM) and at the C-terminus to a V5 epitope tag and the transmembrane domain of glycophorin A, followed by mCherry, C-Myc, and 6xHis tags (Figure 6A). As a control, we used an mCherry construct containing all elements except the B9 or P36 sequences. Codon-optimized versions of the tandem 6-cys domains of *P. berghei* P36 and P52 (amino acids 33-302) were expressed either as transmembrane proteins with 3xFlag and GFP tags or as soluble secreted proteins (sol), with a 3xFlag epitope tag only at the C-terminus (Figure 6A). Following transient transfection of Hepa1-6 cells, all protein constructs distributed mainly intracellularly (likely in the endoplasmic reticulum [ER]), but a fraction was correctly targeted to the cell plasma membrane, as evidenced by immunolabeling of non-permeabilized cells with anti-V5 and anti-Flag antibodies (Figure 6B), suggesting correct folding. Interestingly, the soluble forms of PbP36 (P36-Sol) and PbP52 (P52-Sol) were also detected on the surface of transfected cells, indicating that both are secreted and could interact with host cell membrane factors (Figure 6C). Interaction between proteins was then tested in co-transfection experiments in Hepa1-6 cells, by immunoprecipitation followed by western blot. Both P52-GFP (Figure 6D) and P52-sol (Figure 6E) proteins were co-immunoprecipitated with P36-mCherry but not with the control mCherry protein, validating the strategy and confirming the interaction between *P. berghei* P36 and P52 proteins. More importantly, these experiments showed that P36 and P52 co-immunoprecipitated with B9-mCherry, in both transmembrane (Figure 6D) and soluble (Figure 6E) configurations. Although the interactions were only observed using a heterologous expression system, these results suggest that B9, P36, and P52 may form a supramolecular protein complex. When considering our functional data, such a complex could mediate productive invasion of hepatocytes by sporozoites.

## Discussion

Productive invasion of hepatocytes is a crucial step following transmission of the malaria parasite by a mosquito; however, the molecular mechanisms involved remain poorly understood. Until now, only two sporozoite-specific proteins, the 6-cys proteins P36 and P52, have been associated with productive host cell invasion.^6^^,^^8^ Here we identify another member of the 6-cys family, B9, as a crucial entry factor. Our data confirm that B9 is required for sporozoite infectivity, as reported previously.^15^ However, in that study, the authors concluded that B9 is not expressed in sporozoites and is not involved during parasite entry into hepatocytes. This conclusion was based on an indirect promoter assay in *P. berghei* and immunofluorescence assays in *P. falciparum* using antibodies generated against a 152 aa recombinant protein (233Asn-384Glu), representing a truncated propeller domain that may not reproduce the native protein conformation of the entire PfB9 propeller (26Leu-384Glu). Here, we demonstrate through genetic tagging that B9 is expressed in *P. berghei* sporozoites, corroborating mass spectrometry data.^21^^,^^22^^,^^23^^,^^24^ Furthermore, direct quantification of invasion by flow cytometry established that PbΔ*b9* parasites have an invasion defect. In addition, PbΔ*b9* sporozoites do not discharge their rhoptries upon contact with host cells, similar to PbΔ*p36* sporozoites, indicating that both proteins are acting at an early step during invasion. We further provide evidence that B9 interacts with P36 and P52 using a heterologous expression system, suggesting that the three proteins could participate in an invasion complex required for productive invasion of hepatocytes.

Our data show that two other sporozoite 6-cys proteins, P12p and P38, are dispensable for infection of the liver, in both *P. berghei* and *P. yoelii*. Interestingly, there was a slight delay in the onset of blood-stage patency in mice following inoculation of PbΔ*p12p* or PyΔ*p12p* mutant sporozoites, associated with reduced numbers of PbΔ*p12p* EEFs in HepG2 cell cultures. This suggests that P12p, while nonessential, could nevertheless contribute to optimal sporozoite infection in the liver, a possibility that deserves further investigation.

**Figure 7:**
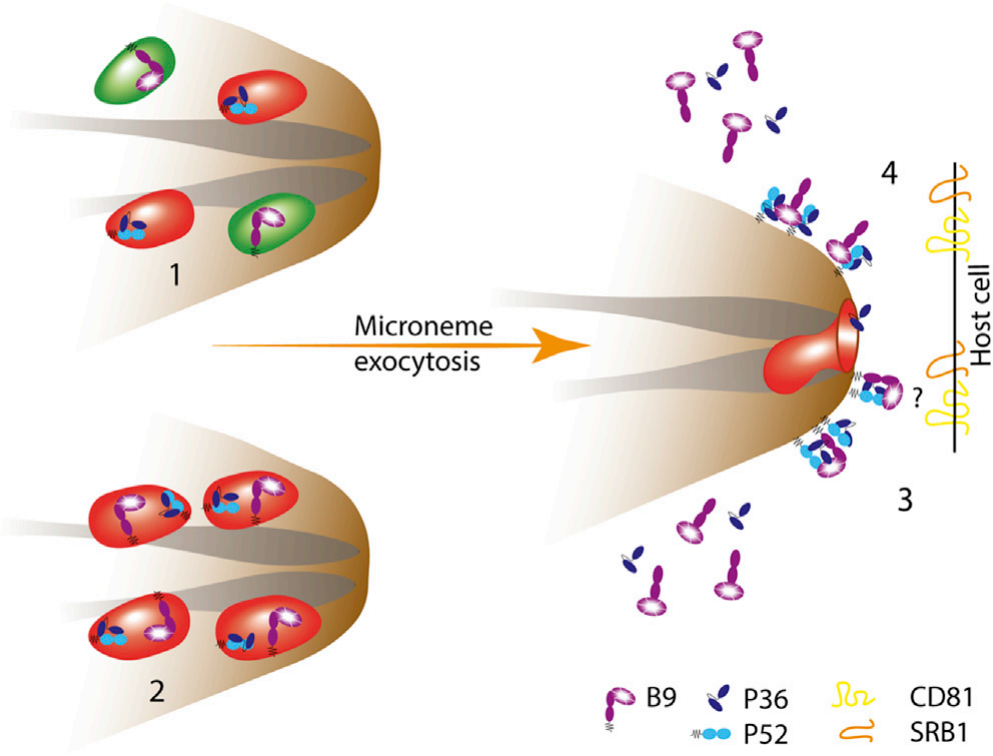
Model for B9 interaction with P36 and P52 for sporozoite host cell invasion B9 may localize in distinct (1) or same (2) micronemes as P36 and P52. Upon microneme exocytosis, the three proteins are released onto the sporozoite surface where B9 could associate with P36 and P52, either as a membrane-bound form (3) or as a processed form (4). The complex may participate in direct or indirect interactions with host cell receptors, including CD81 and SRB1, to trigger commitment to productive host cell invasion.

Comparison of profile hidden Markov models between PfB9 and tertiary structure database identified an N-terminus beta-propeller domain structurally similar to CyRPA, a cysteine-rich protein expressed in *P. falciparum* merozoites, where it forms a protein complex that is essential for invasion of erythrocytes.^40^^,^^41^ Our data suggest that the propeller domain of B9 could directly interact with both P36 and P52. We speculate that blades 3 and 4 of the propeller, which are the most conserved, might be involved in these interactions. Importantly, the interaction of B9 with P36 and P52 was detected using a heterologous expression system but not by coIP from sporozoite protein extracts. Our data are consistent with a previous study performed with *P. yoelii* sporozoites, where P52 but not B9 was identified by mass spectrometry after immunoprecipitation of P36.^11^ We speculate that B9 could interact with P36 and P52 only after parasite activation, similar to CyRPA, which forms a complex with Rh5 and Ripr only at the time of merozoite invasion in *P. falciparum*.^40^ However, our attempts to identify B9-associated proteins in cell culture supernatants failed, possibly due to a lack of sensitivity. Alternatively, the presence of 6-cys domains in the native B9 protein may impact the binding properties of the propeller domain. B9 was secreted from sporozoites upon stimulation of microneme exocytosis, as described previously with P36 in *P. yoelii*.^11^ B9 shedding could be associated with enzymatic processing, as suggested by the differential migration pattern in western blots. This suggests two possible models, where B9 may bind to P36/P52 either as a membrane-bound or as a free form (Figure 7). Using STED super-resolution microscopy, we could visualize individual micronemes in sporozoites. Interestingly, the distribution of B9 partially overlapped that of P36, suggesting that a subset of micronemes may contain both proteins. While a previous immuno-electron microscopy study showed that a fraction of P36 and P52 colocalizes with TRAP in micronemes of *P. yoelii* sporozoites,^11^ we did not observe colocalization of B9 with TRAP or AMA1 in *P. berghei* sporozoites. Our data support the hypothesis that sporozoites contain discrete subsets of micronemes, associated with specific functions.^4^ In line with this hypothesis, Δ*b9 sporozoites* show a similar invasion phenotype to Δ*p36* parasites, without alteration of sporozoite migration (which requires TRAP). Using RON4-mCherry as a rhoptry marker in the invasion assays, we observed that Δ*b9* and Δ*p36* sporozoites do not discharge their rhoptries, suggesting that B9 and P36 act upstream of AMA1-dependent MJ formation. AMA1 is also required upstream of liver infection, during sporozoite invasion of the mosquito salivary glands.^46^ This temporal functional difference between AMA1 and B9 is consistent with the observation that the two proteins are contained in distinct secretory compartments.

*P. berghei* and *P. yoelii* sporozoites use different pathways to invade hepatocytes, with the latter being strictly dependent on CD81, like *P. falciparum.*^5^^,^^7^ Interspecies complementation experiments have shown that P36 (but not P52) is a key determinant of this differential usage of host receptors.^6^ Using a similar approach, we show that the propeller domain of PyB9 can functionally replace the homologous sequence in PbB9, however, without altering host receptor usage. This suggests that the B9 propeller does not directly participate in interaction with host receptors. Rather, we hypothesize that B9 may regulate the trafficking and/or binding of P36 to host cells, possibly by concentrating P36-P52 complexes at the surface of the parasite. In contrast, substituting the PfB9 propeller for the *P. berghei* domain abolished protein function, possibly due to impaired protein expression, as suggested by our western blot data, or as a result of altered interactions with *P. berghei* P36 and/or P52. In this regard, the PfB9 and PbB9 propeller domains show only 48% identity at the amino acid level, versus 90% between PyB9 and PbB9 domains (Figure S8). Our data are consistent with a recent study showing that chimeric *P. berghei* sporozoites where the entire PbB9 has been replaced by PfB9 are not infective.^47^ Reciprocally, the essential role of B9 in assembling invasion complexes with P36 and P52 could also explain why *P. falciparum* and *P. vivax* P36 and P52 failed to compensate for the absence of their counterparts in *P. berghei*^6^ as these proteins may not associate with PbB9 to form functional complexes.

Interestingly, an improved version of the neural network-based model AlphaFold^48^ predicts that the C-terminus portion of B9 is organized in three beta sandwiches rather than two (https://alphafold.ebi.ac.uk/). The structures of these domains and their function remain to be experimentally determined. While our data suggest that B9 6-cys-like domains are not required for interaction with P36 and P52, they might regulate the activity of the propeller and/or participate in interactions with host cell surface molecules.

In conclusion, this study reveals that the 6-Cys protein B9 is required for productive host cell invasion by sporozoites. B9 contains a functionally important beta-propeller domain that is required for proper protein expression and could be involved in the formation of a supramolecular protein complex with P36 and P52. Our results suggest that *Plasmodium* sporozoites and merozoites, despite using distinct sets of parasite and host entry factors, may share common structural modules to assemble protein complexes for invasion of host cells. The complex formed by B9, P36, and P52 proteins may represent a potential target for intervention strategies to prevent the initial stages of malaria liver infection.

### Limitations of the study

One of the limitations of this study is use of the rodent malaria model parasite *P. berghei* to dissect the function of B9 beta-propeller domain through genetic approaches. The propeller domain of *P. falciparum* B9 did not allow proper protein expression and/or function in *P. berghei*; therefore, other approaches will be required to determine the function of this domain in human-infecting malaria parasites. The interactions between the beta-propeller domain of B9 and P36 or P52 were only observed in a heterologous expression system but not with endogenous sporozoite proteins. Such interactions may occur in the parasite in a transient manner, possibly during host cell invasion, which is a rare event and difficult to address experimentally. An additional caveat with our heterologous system is that the bulk of parasite proteins is trapped inside transfected Hepa-16 cells, so we cannot rule out interactions occurring between misfolded proteins in the ER. Finally, the interactions between sporozoite 6-cys proteins and host receptors are not addressed in the study.

## STAR★Methods

### Key resources table

**Table:** 

REAGENT or RESOURCE	SOURCE	IDENTIFIER
**Antibodies**		

Goat polyclonal anti-PbUIS4	Sicgen	Cat#AB0042; RRID: AB_2333158
Mouse monoclonal anti-Flag® (M2 clone)	Sigma-Aldrich	Cat#F3165; RRID: AB_259529
Mouse monoclonal anti-V5	Invitrogen	Cat# R960-25; RRID: AB_2556564
Rat monoclonal anti-mCherry, clone 16D7	Invitrogen	Cat#M11217; RRID: AB_2536611
Rat monoclonal anti-mCD81, clone MT81	Silvie et al., 2006^49^	N/A
Rat monoclonal anti-*Plasmodium* AMA1, clone 28G2	BEI Resources	MRA-897A
Rabbit polyclonal anti-PbTRAP	Klug et al., 2020^50^	N/A
Goat polyclonal anti-GFP	Sicgen	Cat# AB0066-200; RRID: AB_2333101
Rabbit polyclonal anti-GFP	Proteintech	Cat#50430-2-AP; RRID: AB_11042881
Goat anti-mouse IgG AF594	Molecular Probes	Cat#A-11032; RRID: AB_2534091
Donkey anti-rabbit IgG AF594	Molecular Probes	Cat#A-21207; RRID: AB_141637
Goat anti-rat IgG AF594	Molecular Probes	Cat# A-11007; RRID: AB_141374
Goat anti-mouse atto647N	Antibodies-online GmbH	Cat#ABIN964964
Goat anti-mouse STAR RED	Abberior	Cat#STRED-1001
Goat anti-mouse AF680	Thermo Fisher Scientific	Cat#A21058; RRID: AB_2535724
Donkey anti-goat IgG AF680	Thermo Fisher Scientific	Cat#A-21084; RRID: AB_141494
Goat anti-rabbit IgG AF680	Thermo Fisher Scientific	Cat#A-21109; RRID: AB_2535758
Goat anti-mouse IgG DyLight™ 800	Thermo Fisher Scientific	Cat#SA5-3552; RRID: AB_2556774

**Bacterial and virus strains**		

XL1-Blue Competent Cells	Agilent	Cat#200249

**Chemicals, peptides, and recombinant proteins**		

Lipofectamine 2000 reagent	Life Technologies	Cat#11668019; CAS: 158571-62-1
Dextran Tetramethylrhodamine	Invitrogen	Cat#D1817
ProLong™ Diamond Antifade Mountant	Life Technologies	Cat#P36970; RRID: SCR_015961
RFP-Trap Agarose beads	Chromotek	Cat#rta-20
Pyrimethamine	MP Biologicals	Cat# 0219418025 CAS: 58-14-0
Formaldehyde	Electron Microscopy Sciences	Cat#15714 CAS: 50-00-0
Triton X-100	Sigma-Aldrich	Cat#T8787 CAS: 9036-19-5
Hoechst 33342	Invitrogen	Cat#H3570 CAS: 23491-52-3
Igepal® CA-630	Sigma-Aldrich	Cat#56741 CAS: 9002-93-1
Protein G Sepharose™ 4 Fast Flow	Sigma-Aldrich	Cat#17-0618-02
Anti-FLAG®M2 Affinity Gel	Sigma-Aldrich	Cat#A2220

**Critical commercial assays**		

In-Fusion HD Cloning Kit	Clontech	Cat#638911
DNeasy Blood & Tissue Kit	Qiagen	Cat#69504
Plasmid Maxi Kit	Qiagen	Cat#12162
Human T Cell Nucleofector™ Kit	Lonza	Cat#VPA-1002

**Experimental models: Cell lines**		

Mouse: Hepa1-6 hepatic cells	ATCC	ATCC: CRL-1830; RRID: CVCL_0327
Human: HepG2 hepatic cells	ATCC	ATCC: HB-8065; RRID: CVCL_0027
Human: HepG2/CD81 hepatic cells	Silvie et al., 2006^49^	N/A

**Experimental models: Organisms/strains**		

Mouse: C57BL/6JRj	Janvier Labs	N/A
Mouse: BALB/cJRj	Janvier Labs	N/A
Mouse: RjOrl:SWISS (CD-1)	Janvier Labs	N/A
*Anopheles stephensi* mosquitoes	Radboud University Medical Center	N/A
*Plasmodium berghei* ANKA strain, clone 15cy1	BEI Resources	MRA-871
*Plasmodium yoelii* 17XNL strain, clone 1.1	BEI Resources	MRA-593
*Plasmodium berghei* ANKA PbGFP	Manzoni et al., 2014^25^	N/A
*Plasmodium yoelii* 17XNL PyGFP	Manzoni et al., 2014^25^	N/A
*Plasmodium berghei* ANKA PbΔp12p	This study	N/A
*Plasmodium berghei* ANKA PbΔp38	This study	N/A
*Plasmodium berghei* ANKA PbΔb9	This study	N/A
*Plasmodium berghei* ANKA PbΔp36	Manzoni et al., 2017^6^	N/A
*Plasmodium yoelii* 17XNL PyΔp12p	This study	N/A
*Plasmodium yoelii* 17XNL PyΔp38	This study	N/A
*Plasmodium yoelii* 17XNL PyΔb9	This study	N/A
*Plasmodium yoelii* 17XNL PyΔp36	This study	N/A
*Plasmodium berghei* ANKA PbGFP::RON4mCherry	This study	N/A
*Plasmodium berghei* ANKA PbΔb9::RON4mCherry	This study	N/A
*Plasmodium berghei* ANKA PbΔp36::RON4mCherry	This study	N/A
*Plasmodium berghei* ANKA PbB9-Flag	This study	N/A
*Plasmodium berghei* ANKA P36-mCherry	This study	N/A
*Plasmodium berghei* ANKA PbΔb9 complemented PbB9	This study	N/A
*Plasmodium berghei* ANKA PbΔb9 complemented Δprop	This study	N/A
*Plasmodium berghei* ANKA PbΔb9 complemented Δprop6cys1	This study	N/A
*Plasmodium berghei* ANKA PbΔb9 complemented Pyprop	This study	N/A
*Plasmodium berghei* ANKA PbΔb9 complemented Pyprop6cys1	This study	N/A
*Plasmodium berghei* ANKA PbΔb9 complemented Pfprop	This study	N/A
*Plasmodium berghei* ANKA PbΔb9 complemented Δprop-Flag	This study	N/A
*Plasmodium berghei* ANKA PbΔb9 complemented Pfprop-Flag	This study	N/A

**Oligonucleotides**		

Primers for plasmids constructs and parasite genotyping, see Table S4	This paper	N/A

Recombinant DNA		

GOMO-GFP plasmid	Manzoni et al., 2014^25^	Addgene Plasmid #60975
B3D + mCherry plasmid	Silvie et al., 2008^51^	N/A
pCEN-SPECT2 plasmid	Iwanaga et al., 2010^52^	N/A
pEF1α-AcGFP1-N1 plasmid	Clontech	Cat# 631973

**Software and algorithms**		

ImageJ	Schneider et al., 2012^53^	https://ImageJ.nih.gov/ij/
PSIPRED 4.0	Buchan and Jones, 2019^54^	http://bioinf.cs.ucl.ac.uk/psipred/
Inter-Pro	Mulder et al., 2002^55^	https://www.ebi.ac.uk/interpro/
HHpred	Söding et al., 2005^56^	https://toolkit.tuebingen.mpg.de/tools/hhpred
NetGPI tool	Gíslason et al., 2021^57^	https://services.healthtech.dtu.dk/service.php?NetGPI
IUPred2A web server	Mészáros et al., 2018^58^	https://iupred2a.elte.hu/
Robetta web server	The Baker lab	https://robetta.bakerlab.org/
GalaxyRefine	Heo et al., 2013^59^	https://bio.tools/galaxyrefine
Yasara	Krieger et al., 2009^60^	http://www.yasara.org/
MolProbity	Chen et al., 2010^61^	http://molprobity.manchester.ac.uk/
Prosa II	Wiederstein and Sippl, 2007^62^	https://prosa.services.came.sbg.ac.at/prosa.php
UCSF Chimera	Pettersen et al., 2004^63^	https://www.cgl.ucsf.edu/chimera/
Adaptive Poisson-Boltzmann Solver	Baker et al., 2001^64^	https://mccammon.ucsd.edu/iapbs/
PDB2PQR v.2.1.1	Dolinsky et al., 2004^65^	https://www.cgl.ucsf.edu/chimera/docs/ContributedSoftware/apbs/pdb2pqr.html
MAFFT version 7	Katoh and Standley, 2013^66^	https://mafft.cbrc.jp/alignment/server/
PhyML v3.0	Guindon et al., 2010^67^	http://www.atgc-montpellier.fr/phyml/
Smart Model Selection (SMS)	Lefort et al., 2017^68^	http://www.atgc-montpellier.fr/sms/
aLRT SH-like method	Anisimova et al., 2001^69^	http://www.atgc-montpellier.fr/phyml/alrt/
GP4Rate tool: FuncPatch *(now offline)*	Huang and Golding, 2014^70^	http://info.mcmaster.ca/yifei/FuncPatch/
X!Tandem pipeline (version 0.2.36)	Langella et al., 2017^71^	https://www.thegpm.org/TANDEM/
R version 3.5.2	R Foundation for Statistical Computing	https://www.r-project.org/
GraphPad Prism 7	GraphPad Software	https://www.graphpad.com/scientific-software/prism/
Huygens Professional Deconvolution software v18.10	Scientific Volume Imaging	https://svi.nl/Huygens-Essential

### Resource availability

#### Lead contact

Further information and requests for resources and reagents should be directed to and will be fulfilled by the lead contact, Olivier Silvie (olivier.silvie@inserm.fr).

#### Materials availability

All unique reagents generated in this study are available upon reasonable request from the lead contact, Olivier Silvie (olivier.silvie@inserm.fr). The GOMO-GFP plasmid is available from Addgene (#60975).

### Experimental model and subject details

#### Ethics Statement

All mouse work was conducted in strict accordance with the Directive 2010/63/EU of the European Parliament and Council ‘On the protection of animals used for scientific purposes’. Protocols were approved by the Ethical Committee Charles Darwin N 005 (approval #7475-2016110315516522).

#### Experimental animals, parasites, and cell lines

*P. berghei* and *P. yoelii* blood stage parasites were propagated in female Swiss mice (6–8 weeks old, from Janvier Labs). We used wild type *P. berghei* (ANKA strain, clone 15cy1) and *P. yoelii* (17XNL strain, clone 1.1), and GFP-expressing PyGFP and PbGFP parasite lines, obtained after integration of a GFP expression cassette at the dispensable *p230p* locus.^25^ *Anopheles stephensi* mosquitoes were fed on *P. berghei* or *P. yoelii*-infected mice using standard methods,^72^ and kept at 21°C and 24°C, respectively. *P. berghei* and *P. yoelii* sporozoites were collected from the salivary glands of infected mosquitoes 21–28 or 14–18 days post-feeding, respectively. *P. berghei* and *P. yoelii* sporozoite infections were performed in female C57BL/6 or BALB/c mice, respectively (6 weeks old, from Janvier Labs), by intravenous injection in a tail vein. HepG2 (ATCC HB-8065), HepG2/CD81^38^ and Hepa1-6 cells (ATCC CRL-1830) were cultured at 37°C under 5% CO_2_ in DMEM supplemented with 10% fetal calf serum and antibiotics (Life Technologies), as described.^7^ HepG2 and HepG2/CD81 were cultured in culture dishes coated with rat tail collagen I (Becton-Dickinson).

### Method details

#### Gene deletion of *p12p*, *p38* and *b9* in *P. berghei* and *P. yoelii*

Gene deletion mutant parasites were generated using our ‘‘Gene Out Marker Out’’ (GOMO) strategy.^25^ For each target gene, a 5’ fragment and a 3’ fragment were amplified by PCR from *P. berghei* (ANKA) or *P. yoelii* (17XNL) WT genomic DNA, using primers listed in Table S4, and inserted into *Sac*II/*Not*I and *Xho*I/*Kpn*I restriction sites, respectively, of the GOMO-GFP vector,^25^ using the In-Fusion HD Cloning Kit (Clontech). The resulting targeting constructs were linearized with *Sac*II and *Kpn*I before transfection. All constructs used in this study were verified by DNA sequencing (Eurofins Genomics). Purified schizonts of *P. berghei* ANKA or *P. yoelii* 17XNL WT parasites were transfected with targeting constructs by electroporation using the AMAXA Nucleofector^TM^ device, as described,^73^ and immediately injected intravenously in mice. GFP-expressing parasite mutants were then isolated by flow cytometry after positive and negative selection rounds, as described.^25^ Parasite genomic DNA was extracted using the DNeasy Blood & Tissue Kit (Qiagen), and analyzed by PCR using primer combinations specific for WT, 5’ or 3’ recombined and marker excised loci (listed in Table S4).

#### Genetic tagging of RON4, P36 and B9

Fusion of mCherry at the C-terminus of RON4 was achieved through double crosser homologous recombination. For this purpose, 5’ and 3’ homology fragments, consisting of a 1.2 kb terminal RON4 fragment (immediately upstream of the stop codon) and a 0.6 kb downstream fragment were amplified by PCR using primers listed in Table S4, and cloned into *Not*I/*Spe*I and *Hind*III/*Kpn*I sites, respectively, of the B3D+mCherry plasmid.^51^ The resulting construct was linearized with *Not*I and *Kpn*I before transfection of PbGFP, PbΔ*b9* or PbΔ*p36* purified schizonts. Recombinant parasites were selected with pyrimethamine and cloned by limiting dilution and injection into mice. Integration of the construct was confirmed by PCR on genomic DNA using specific primer combinations listed in Table S4. P36 fused with mCherry at the C-terminus was expressed from a centromeric episomal plasmid. For this purpose, we first introduced *ama1* promoter and 3’ UTR fragments in the centromeric pCEN-SPECT2 plasmid,^52^ between *Kpn*I and *Sal*I sites. In a second step, we introduced P36 and mCherry inserts in the *Nde*I site of the plasmid. The resulting construct was used to transfect marker-free PbΔ*p36* parasites. Parasites harboring the P36-mCherry stable episomal construct were isolated after a single round of selection with pyrimethamine. Tagging of *P. berghei* B9 with a triple Flag epitope was achieved by double crossover homologous recombination with the endogenous *B9* gene locus. For this purpose, three inserts were amplified by PCR and sequentially inserted in two steps using the In-Fusion HD Cloning Kit (Clontech). In the first step, a 3’ homology 736-bp fragment was cloned into the *Nhe*I site in a plasmid containing a GFP-2A-hDHFR cassette under control of the *P. yoelii* HSP70 promoter. In the second step, a 5’ homology 759-bp fragment from B9 ORF and a 789-bp fragment comprising a triple Flag epitope, a recodonized B9 C-terminus sequence and the 3’ UTR of PyB9 were inserted into *Kpn*I/*Eco*RI sites of the plasmid. Primers used to assemble the B9 tagging construct and the sequence of the synthetic gene are listed in Table S4. The resulting construct was linearized with *Kpn*I and *Nhe*I before transfection of WT *P. berghei* (ANKA) parasites. Recombinant parasites were selected with pyrimethamine and cloned by limiting dilution and injection into mice. Integration of the construct was confirmed by PCR on genomic DNA using specific primer combinations listed in Table S4. To obtain sporozoites expressing tagged versions of both B9 and P36, we performed a genetic cross between B9-Flag and P36-mCherry parasites. For this purpose, mice were injected with equal amounts of B9-Flag and P36-mCherry-infected erythrocytes and used for transmission to mosquitoes.

#### Structure-guided mutagenesis of *P. berghei* B9

Genetic complementation of PbΔ*b9* parasites was achieved by double crossover homologous recombination using a vector containing a hDHFR cassette and a 3’ homology arm corresponding to the 5’ sequence of the HSP70 promoter of the GFP cassette in the PbΔ*b9* line. First, an 840-bp fragment including the coding sequence for PbB9 N-terminus (amino acids 1-29), and a 1096-bp fragment encoding the C-terminus (amino acids 647-852) followed by the 3’ UTR of PbB9 were sequentially inserted into the plasmid, in *Kpn*I/*Eco*RI sites, resulting in the ΔpropΔ6cys1 construct. Cloning of a 1950-bp fragment of PbB9 gene (including the coding sequence for amino acids 30-646) into *Xho*I/*Kpn*I sites of the ΔpropΔ6cys1 plasmid resulted in the PbB9 construct, encoding the full length PbB9 protein. Cloning of a 912-bp fragment of PbB9 gene (including the coding sequence for amino acids 344-646) into *Xho*I/*Kpn*I sites of the ΔpropΔ6cys1 plasmid resulted in the Δprop construct. Cloning of a 1992-bp fragment from PyB9 gene (including the coding sequence for amino acids 30-653 of PyB9) into *Xho*I/*Kpn*I sites of the ΔpropΔ6cys1 plasmid resulted in the PyProp6cys1 construct. Cloning of a 948-bp fragment from PyB9 gene (encoding amino acids 30-342 of PyB9) and a 903-bp fragment from PbB9 gene (encoding amino acids 346-646 of PbB9) into *Xho*I/*Kpn*I sites of the ΔpropΔ6cys1 plasmid resulted in the PyProp construct. Cloning of a 1071-bp fragment from PfB9 gene (encoding amino acids 25-379 of PfB9) and a 903-bp fragment from PbB9 gene (encoding amino acids 346-646 of PbB9) into *Xho*I/*Kpn*I sites of the ΔpropΔ6cys1 plasmid resulted in the PfProp construct. Two additional constructs were generated encoding Δprop and PfProp variants containing a 3xFlag epitope. For this purpose, we first assembled a ΔpropΔ6cys_Flag plasmid containing a 840-bp fragment including the coding sequence for PbB9 N-terminus (amino acids 1-29) and a 795-bp fragment amplified from the B9 tagging construct and corresponding to Flag-tagged PbB9 C-terminus and PyB9 3’ UTR. Subsequent cloning of a 1266-bp fragment of PbB9 gene (encoding amino acids 351-772) into *Xho*I/*Kpn*I sites of the ΔpropΔ6cys_Flag plasmid resulted in the Δprop_Flag construct. In parallel, a 2337-bp fragment corresponding to the PfB9 propeller domain followed by PbB9 6cys domains was amplified from the PfProp construct and inserted into *Xho*I/*Kpn*I sites of the ΔpropΔ6cys_Flag plasmid, resulting in the PfProp_Flag construct. The primers used to assemble the constructs for genetic complementation are listed in Table S4. The constructs were linearized with *Nhe*I before transfection of PbΔ*b9* purified schizonts. Recombinant parasites were selected with pyrimethamine. Integration of the constructs was confirmed by PCR on genomic DNA using specific primer combinations listed in Table S4.

#### Sporozoite invasion assays

Host cell invasion by GFP-expressing sporozoites was monitored by flow cytometry.^74^ Briefly, hepatoma cells (3 × 10^4^ per well in collagen-coated 96-well plates) were incubated with sporozoites (5 × 10^3^ to 1 × 10^4^ per well). For measurement of cell traversal activity, sporozoites were incubated with cells in the presence of 0.5 mg/ml rhodamine-conjugated dextran (Life Technologies). At different time points ranging from 15 minutes to 3 hours post-infection, cell cultures were washed, trypsinized and analyzed on a Guava EasyCyte 6/2L bench cytometer equipped with 488 nm and 532 nm lasers (Millipore), for detection of GFP-positive cells and dextran-positive cells, respectively, to measure total invasion rates and cell traversal activity. To assess liver stage development, HepG2 or HepG2/CD81 cells were infected with GFP-expressing sporozoites and cultured for 24-36 hours before analysis either by FACS or by fluorescence microscopy, after fixation with 4% Formaldehyde (FA) and labeling with antibodies specific for UIS4 (Sicgen).

#### Fluorescence microscopy

To visualize RON4-mCherry in transgenic parasites, purified schizonts and sporozoites were deposited on poly-L-lysine coated coverslips and fixed with 4% FA. GFP and mCherry images were captured on a Zeiss Axio Observer.Z1 fluorescence microscope equipped with a Plan- Apochromat 63×/1.40 Oil DIC M27 objective. Images acquired using the Zen 2012 software (Zeiss) were processed with ImageJ^53^ or Photoshop CS6 software (Adobe) for adjustment of contrast. To quantify rhoptry discharge, RON4-mCherry expressing PbGFP, PbΔ*b9* or PbΔ*p36* sporozoites were incubated with HepG2 cells for 3 h at 37°C. After extensive washes to remove extracellular parasites, cultures were trypsinized and cells were examined under a fluorescence microscope to assess for mCherry fluorescence in GFP-expressing intracellular sporozoites. At least 50 intracellular parasites in triplicate wells were examined for each parasite line. The percentage of rhoptry discharge was defined as the proportion of intracellular sporozoites without detectable RON4-mCherry signal. For immunofluorescence analysis of Flag-tagged parasites, sporozoites collected from infected mosquito salivary glands were deposited on poly-L-lysine coated coverslips, fixed with 4% FA and permeabilized with 1% Triton X-100. Parasites were labelled with anti-Flag mouse antibodies (M2 clone, Sigma) and AlexaFluor 594-conjugated secondary antibodies (Life Technologies). Nuclei were stained with Hoechst 33342. For double labelling of B9 and AMA1, we used anti-Flag mouse antibodies (M2 clone, Sigma) and anti-AMA1 rat antibodies^75^ (clone 28G2, Bei Resources), followed by atto647N-conjugated anti-mouse and Alexa-594-conjugated anti-rat antibodies. For double labelling of B9 and P36-mCherry or TRAP, we used anti-Flag mouse antibodies (M2 clone, Sigma), anti-mCherry rat antibodies (Invitrogen) or anti-TRAP rabbit antibodies,^50^ followed by STAR RED-conjugated anti-mouse and Alexa-594-conjugated anti-rat antibodies or atto647N-conjugated anti-mouse and Alexa-594-conjugated anti-rabbit antibodies, respectively. Coverslips were mounted on glass slides with ProLong™ Diamond Antifade Mountant (Life Technologies). STED imaging was carried out with a 93x glycerol-immersion objective (NA 1.3) on a Leica TCS SP8 STEDX microscope equipped with a White Light Laser. AlexaFluor 594 and atto647N- or STAR RED-labelled compartments were excited at 590 or 644 nm, respectively, and depleted with a pulsed 775 nm STED laser. Image frames were acquired sequentially frame by frame at a scan speed of 200 lines/s with an optimal pixel size and a line average of 4 to 8. Deconvolution of STED data was performed using the default deconvolution settings in Huygens Professional Deconvolution software v18.10 (Scientific Volume Imaging) that were estimated from the metadata. Brightness and Contrast were adjusted using Fiji.^76^

#### Western blot

B9-Flag sporozoites were isolated from the salivary glands of infected mosquitoes and resuspended in 1X PBS. Microneme secretion was stimulated by incubation for 15 min at 37°C in a buffer containing 1% BSA and 1% ethanol, as described.^77^ Pellet and supernatant fractions were then isolated from activated and non-activated (control) sporozoites, resuspended in Laemmli buffer and analyzed by SDS-PAGE under non-reducing conditions. For the Δprop-Flag and Pfprop-Flag parasites, only pellet fractions were analyzed. Western blotting was performed using primary antibodies against the Flag epitope (M2 clone, Sigma) or against GFP (loading control), and secondary antibodies coupled with Alexa Fluor 680. Membranes were then analyzed using the InfraRed Odyssey system (Licor).

#### Heterologous expression of *Plasmodium* proteins in Hepa1-6 cells

Two vectors for mammalian cell expression were first assembled in a pEF1α-AcGFP1-N1 backbone. The first one (mCherry) encodes a cassette consisting of the signal peptide from bee venom melittin (BVM), a V5 epitope, the transmembrane and C-terminus of mouse Glycophorin A (GYPA), mCherry, Myc and 6xHis tags. In the second one (GFP), the cassette encodes the signal peptide from BVM, a 3xFlag epitope, the transmembrane and C-terminus of mouse GYPA, and GFP. Codon-optimized versions of PbB9 propeller domain (amino acids 31-348), PbP36 (amino acids 67-352) or PbP52 (amino acids 33-302) were inserted in the mCherry and/or GFP plasmids between the signal peptide and the Flag or V5 epitope tag. Two additional constructs for expression of soluble PbP36 and PbP52 were obtained by adding a stop codon immediately after the 3xFlag epitope. The construct cassette sequences are indicated in Table S5. High concentration plasmid solutions were produced using XL1-Blue Competent Cells (Agilent) and plasmid extraction was performed using Qiagen Plasmid Maxikit (Qiagen) according to the manufacturer’s recommendations. Plasmid transfection was performed in Hepa1-6 cells using the Lipofectamine 2000 reagent (Life Technologies) according to the manufacturer’s specifications. Following plasmid transfection, cells were cultured for 24 h before lysis in a buffer containing 1% NP40 (Igepal CA-630). Protein extracts were then subjected to immunoprecipitation using agarose beads coupled with anti-RFP nanobodies (Chromotek). Eluates were collected and analyzed by western blot, using anti-Flag antibodies. Membranes were analyzed using the InfraRed Odyssey system (Licor). Expression of *P. berghei* proteins in Hepa1-6 cells was also analyzed by fluorescence microscopy, after fixation of transfected cells with 4% FA (without permeabilization) and staining with anti-Flag or anti-V5 antibodies, together with the anti-mouse CD81 MT81 rat monoclonal antibody^49^ and Hoechst 33342, to label the host cell membranes and nuclei, respectively.

#### B9 immunoprecipitation and mass spectrometry

Freshly dissected B9-Flag sporozoites were lysed on ice for 30 min in a lysis buffer containing 0.5% w/v NP40 (Igepal CA-630) and protease inhibitors. After centrifugation (15,000 × g, 15 min, 4°C), supernatants were incubated with protein G-conjugated sepharose for preclearing overnight. Precleared lysates were subjected to B9-Flag immunoprecipitation using Anti-FLAG M2 Affinity Gel (Sigma) for 2h at 4°C, according to the manufacturer's protocol. PbGFP parasites with untagged B9 were used as a control and treated in the same fashion. After washes, proteins on beads were eluted in 2X Laemmli and denatured (95°C, 5min). After centrifugation, supernatants were collected for further analysis. Samples were subjected to a short SDS-PAGE migration, and gel pieces were processed for protein trypsin digestion by the DigestProMSi robot (Intavis), as described.^21^ Peptide samples were analyzed on a timsTOF PRO mass spectrometer (Bruker) coupled to the nanoElute HPLC, as described.^21^ Mascot generic files were processed with X!Tandem pipeline (version 0.2.36)^71^ using the PlasmoDB_PB_39_PbergheiANKA database, as described.^21^ The mass spectrometry proteomics data have been deposited to the ProteomeXchange Consortium via the PRIDE^78^ partner repository with the dataset identifier PXD034830.

#### Structural analyses of B9 propeller

The secondary structure of PfB9 was predicted by hydrophobic cluster analysis^79^ and using PSIPRED 4.0.^54^ Conserved domains were searched using InterPro^55^ and HHpred.^56^ Glycosylphosphatidylinositol (GPI) anchors were predicted using the NetGPI tool (https://services.healthtech.dtu.dk/service.php?NetGPI).^57^ Intrinsic disorder prediction was made using the IUPred2A web server (https://iupred2a.elte.hu/).^58^ The homology model of PfB9 propeller (amino acids 26 to 386) was built with the X-ray structure at 2.4 Å resolution of CyRPA from *P. falciparum* (PDB ID: 5TIH^42^) using the Robetta web server (https://robetta.bakerlab.org/, default parameters). The model was refined and energy-minimized using respectively GalaxyRefine^59^ and Yasara,^60^ then validated using MolProbity^61^ and Prosa II^62^ (Figure S7). Structural alignment of PfB9 propeller and CyRPA was performed using the *MatchMaker* function in UCSF Chimera.^63^ Protein electrostatic surface potential was calculated using Adaptive Poisson-Boltzmann Solver (APBS^64^), after determining the per-atom charge and radius of the structure with PDB2PQR v.2.1.1.^65^ The Poisson-Boltzmann equation was solved at 298 K using a grid-based method, with solute and solvent dielectric constants fixed at 2 and 78.5, respectively. We used a scale of −5 *kT/e* to +5 *kT/e* to map the electrostatic surface potential in a radius of 1.4 Å. All tertiary structures were visualized and drawn using UCSF Chimera.^63^

#### Evolutionary analysis of B9 and CyRPA

The amino acid sequence of PfB9 (PlasmoDB code: PF3D7_0317100) and CyRPA (PF3D7_0423800) were queried against the PlasmoDB database^80^ (release 46) and the NCBI non-redundant protein database using blastp searchs (BLOSUM62 scoring matrix). Twenty-three B9 and eighteen CyRPA sequences were retrieved from distinct *Plasmodium* species. Protein sequence alignments were generated using MAFFT version 7 (default parameters^66^). Output alignments were visually inspected and manually edited with BioEdit v7.2.5. Amino acid positions containing gaps in at least 30% of all sequences were removed. Phylogenetic relationships of B9 and CyRPA amino acid sequences were inferred using the maximum-likelihood method implemented in PhyML v3.0,^67^ after determining the best-fitting substitution model using the Smart Model Selection (SMS) package.^68^ The nearest neighbour interchange approach was chosen for tree improving, and branch supports were estimated using the approximate likelihood ratio aLRT SH-like method.^69^ Site-specific substitution rates were estimated by considering their spatial correlation in tertiary structure using the GP4Rate tool.^70^ GP4rate requires an amino acid sequence alignment, a phylogenetic tree and a protein tertiary structure to estimate the conservation level during species evolution and the characteristic length scale (in Å) of spatially correlated site-specific substitution rates. For B9, we used the refined tertiary structure predicted by Robetta, while we chose the X-ray structure resolved at 2.4 Å resolution for CyRPA (PDB ID: 5TIH^42^).

### Quantification and statistical analysis

Statistical significance of infection data was assessed by one-way ANOVA followed by Dunnett’s multiple comparisons test, two-way ANOVA followed by Sidak’s multiple comparisons test, or two-tailed ratio paired t test, as indicated in the figure legends. Survival curves were analyzed using the Log rank Mantel-Cox test. All statistical tests were computed with GraphPad Prism 7 (GraphPad Software). *In vitro* experiments were performed with a minimum of three technical replicates per experiment. Quantitative source data are provided in Table S6. Statistical analyses for structural modelling were performed using the computing environment R version 3.5.2 (R Foundation for Statistical Computing). For all statistical tests, mean values were regarded as significantly different at p < 0.05.

## Data Availability

• This paper does not report original code. • All quantitative data are provided in Table S6. • Any additional information required to reanalyze the data reported in this paper is available from the lead contact upon request.

## Supplemental information

Document S1. Figures S1–S11

Table S1. List of Plasmodium B9 proteins used for evolutionary analysis, related to STAR Methods

Table S2. Site-specific substitution rates in PfB9, related to STAR Methods

Table S3. *P. berghei* proteins identified by mass spectrometry after anti-Flag immunoprecipitation, related to STAR Methods

Table S4. List of primers used in the study, related to STAR Methods

Table S5. Construct sequences for heterologous expression of proteins in mammalian cells, related to STAR Methods

Table S6. Quantitative source data, related to STAR Methods

## References

[1] World Health Organization (2021).

[2] RTSS Clinical Trials Partnership (2015). Efficacy and safety of RTS, S/AS01 malaria vaccine with or without a booster dose in infants and children in Africa: final results of a phase 3, individually randomised, controlled trial. Lancet.

[3] Risco-Castillo V., Topçu S., Marinach C., Manzoni G., Bigorgne A.E., Briquet S., Baudin X., Lebrun M., Dubremetz J.F., Silvie O. (2015). Malaria sporozoites traverse host cells within transient vacuoles. Cell Host Microbe.

[4] Loubens M., Vincensini L., Fernandes P., Briquet S., Marinach C., Silvie O. (2021). Plasmodium sporozoites on the move: switching from cell traversal to productive invasion of hepatocytes. Mol. Microbiol..

[5] Silvie O., Rubinstein E., Franetich J.F., Prenant M., Belnoue E., Rénia L., Hannoun L., Eling W., Levy S., Boucheix C., Mazier D. (2003). Hepatocyte CD81 is required for Plasmodium falciparum and Plasmodium yoelii sporozoite infectivity. Nat. Med..

[6] Manzoni G., Marinach C., Topçu S., Briquet S., Grand M., Tolle M., Gransagne M., Lescar J., Andolina C., Franetich J.F. (2017). Plasmodium P36 determines host cell receptor usage during sporozoite invasion. Elife.

[7] Silvie O., Franetich J.F., Boucheix C., Rubinstein E., Mazier D. (2007). Alternative invasion pathways for plasmodium berghei sporozoites. Int. J. Parasitol..

[8] Ishino T., Chinzei Y., Yuda M. (2005). Two proteins with 6-cys motifs are required for malarial parasites to commit to infection of the hepatocyte. Mol. Microbiol..

[9] Labaied M., Harupa A., Dumpit R.F., Coppens I., Mikolajczak S.A., Kappe S.H.I. (2007). Plasmodium yoelii sporozoites with simultaneous deletion of P52 and P36 are completely attenuated and confer sterile immunity against infection. Infect. Immun..

[10] van Schaijk B.C.L., Janse C.J., van Gemert G.J., van Dijk M.R., Gego A., Franetich J.F., van de Vegte-Bolmer M., Yalaoui S., Silvie O., Hoffman S.L. (2008). Gene discruption of Plasmodium falcifarum p52 results in attenuation of malaria liver stage development in cultured primary human hepatocytes. PLoS One.

[11] Arredondo S.A., Swearingen K.E., Martinson T., Steel R., Dankwa D.A., Harupa A., Camargo N., Betz W., Vigdorovich V., Oliver B.G. (2018). The micronemal plasmodium proteins P36 and P52 act in concert to establish the replication-permissive compartment within infected hepatocytes. Front. Cell. Infect. Microbiol..

[12] Kaushansky A., Douglass A.N., Arang N., Vigdorovich V., Dambrauskas N., Kain H.S., Austin L.S., Sather D.N., Kappe S.H.I. (2015). Malaria parasites target the hepatocyte receptor EphA2 for successful host infection. Science.

[13] Langlois A.C., Marinach C., Manzoni G., Silvie O. (2018). Plasmodium sporozoites can invade hepatocytic cells independently of the ephrin receptor A2. PLoS One.

[14] Arredondo S.A., Kappe S.H.I. (2017). The s48/45 six-cysteine proteins: mediators of interaction throughout the Plasmodium life cycle. Int. J. Parasitol..

[15] Annoura T., Van Schaijk B.C.L., Ploemen I.H.J., Sajid M., Lin J.W., Vos M.W., Dinmohamed A.G., Inaoka D.K., Rijpma S.R., Van Gemert G.J. (2014). Two Plasmodium 6-Cys family-related proteins have distinct and critical roles in liver-stage development. FASEB J.

[16] Taechalertpaisarn T., Crosnier C., Bartholdson S.J., Hodder A.N., Thompson J., Bustamante L.Y., Wilson D.W., Sanders P.R., Wright G.J., Rayner J.C. (2012). Biochemical and functional analysis of two plasmodium falciparum blood-stage 6-Cys proteins: P12 and P41. PLoS One.

[17] Kennedy A.T., Schmidt C.Q., Thompson J.K., Weiss G.E., Taechalertpaisarn T., Gilson P.R., Barlow P.N., Crabb B.S., Cowman A.F., Tham W.-H. (2016). Recruitment of factor H as a novel complement evasion strategy for blood-stage plasmodium falciparum infection. J. Immunol..

[18] Kumar N. (1987). Target antigens of malaria transmission blocking immunity exist as a stable membrane bound complex. Parasite Immunol..

[19] Simon N., Scholz S.M., Moreira C.K., Templeton T.J., Kuehn A., Dude M.A., Pradel G. (2009). Sexual stage adhesion proteins form multi-protein complexes in the malaria parasite Plasmodium falciparum. J. Biol. Chem..

[20] Molina-Cruz A., Canepa G.E., Alves E Silva T.L., Williams A.E., Nagyal S., Yenkoidiok-Douti L., Nagata B.M., Calvo E., Andersen J., Boulanger M.J., Barillas-Mury C. (2020). Plasmodium falciparum evades immunity of anopheline mosquitoes by interacting with a Pfs47 midgut receptor. Proc. Natl. Acad. Sci. USA.

[21] Hamada S., Pionneau C., Parizot C., Silvie O., Chardonnet S., Marinach C. (2021). In-depth proteomic analysis of Plasmodium berghei sporozoites using trapped ion mobility spectrometry with parallel accumulation-serial fragmentation. Proteomics.

[22] Lasonder E., Janse C.J., Van Gemert G.J., Mair G.R., Vermunt A.M.W., Douradinha B.G., Van Noort V., Huynen M.A., Luty A.J.F., Kroeze H. (2008). Proteomic profiling of Plasmodium sporozoite maturation identifies new proteins essential for parasite development and infectivity. PLoS Pathog..

[23] Lindner S.E., Swearingen K.E., Harupa A., Vaughan A.M., Sinnis P., Moritz R.L., Kappe S.H.I. (2013). Total and putative surface proteomics of malaria parasite salivary gland sporozoites. Mol. Cell. Proteomics.

[24] Swearingen K.E., Lindner S.E., Flannery E.L., Vaughan A.M., Morrison R.D., Patrapuvich R., Koepfli C., Muller I., Jex A., Moritz R.L. (2017). Proteogenomic analysis of the total and surface-exposed proteomes of Plasmodium vivax salivary gland sporozoites. PLoS Negl. Trop. Dis..

[25] Manzoni G., Briquet S., Risco-Castillo V., Gaultier C., Topçu S., Ivănescu M.L., Franetich J.F., Hoareau-Coudert B., Mazier D., Silvie O. (2014). A rapid and robust selection procedure for generating drug-selectable marker-free recombinant malaria parasites. Sci. Rep..

[26] Swearingen K.E., Lindner S.E., Shi L., Shears M.J., Harupa A., Hopp C.S., Vaughan A.M., Springer T.A., Moritz R.L., Kappe S.H.I., Sinnis P. (2016). Interrogating the Plasmodium Sporozoite Surface: Identification of Surface-Exposed Proteins and Demonstration of Glycosylation on CSP and TRAP by Mass Spectrometry-Based Proteomics. PLoS Pathog..

[27] Treeck M., Sanders J.L., Elias J.E., Boothroyd J.C. (2011). The Phosphoproteomes of Plasmodium falciparum and Toxoplasma gondii Reveal Unusual Adaptations Within and Beyond the Parasites’ Boundaries. Cell Host Microbe.

[28] Oehring S.C., Woodcroft B.J., Moes S., Wetzel J., Dietz O., Pulfer A., Dekiwadia C., Maeser P., Flueck C., Witmer K. (2012). Organellar proteomics reveals hundreds of novel nuclear proteins in the malaria parasite Plasmodium falciparum. Genome Biol..

[29] Lasonder E., Rijpma S.R., van Schaijk B.C.L., Hoeijmakers W.A.M., Kensche P.R., Gresnigt M.S., Italiaander A., Vos M.W., Woestenenk R., Bousema T. (2016). Integrated transcriptomic and proteomic analyses of P. falciparum gametocytes: molecular insight into sex-specific processes and translational repression. Nucleic Acids Res..

[30] Pease B.N., Huttlin E.L., Jedrychowski M.P., Talevich E., Harmon J., Dillman T., Kannan N., Doerig C., Chakrabarti R., Gygi S.P., Chakrabarti D. (2013). Global analysis of protein expression and phosphorylation of three stages of Plasmodium falciparum intraerythrocytic development. J. Proteome Res..

[31] Silvestrini F., Lasonder E., Olivieri A., Camarda G., van Schaijk B., Sanchez M., Younis Younis S., Sauerwein R., Alano P. (2010). Protein export marks the early phase of gametocytogenesis of the human malaria parasite Plasmodium falciparum. Mol. Cell. Proteomics..

[32] Khan S.M., Franke-Fayard B., Mair G.R., Lasonder E., Janse C.J., Mann M., Waters A.P. (2005). Proteome Analysis of Separated Male and Female Gametocytes Reveals Novel Sex-Specific Plasmodium Biology. Cell.

[33] Lindner S.E., Swearingen K.E., Shears M.J., Walker M.P., Vrana E.N., Hart K.J., Minns A.M., Sinnis P., Moritz R.L., Kappe S.H.I. (2019). Transcriptomics and proteomics reveal two waves of translational repression during the maturation of malaria parasite sporozoites. Nat. Commun..

[34] van Dijk M.R., van Schaijk B.C.L., Khan S.M., van Dooren M.W., Ramesar J., Kaczanowski S., van Gemert G.-J., Kroeze H., Stunnenberg H.G., Eling W.M. (2010). Three members of the 6-cys protein family of Plasmodium play a role in gamete fertility. PLoS Pathog..

[35] Bushell E., Gomes A.R., Sanderson T., Anar B., Girling G., Herd C., Metcalf T., Modrzynska K., Schwach F., Martin R.E. (2017). Functional Profiling of a Plasmodium Genome Reveals an Abundance of Essential Genes. Cell.

[36] Mueller A.K., Labaied M., Kappe S.H.I., Matuschewski K. (2005). Genetically modified Plasmodium parasites as a protective experimental malaria vaccine. Nature.

[37] Ploemen I.H.J., Croes H.J., van Gemert G.J.J., Wijers-Rouw M., Hermsen C.C., Sauerwein R.W. (2012). Plasmodium berghei Δp52&p36 Parasites Develop Independent of a Parasitophorous Vacuole Membrane in Huh-7 Liver Cells. PLoS One.

[38] Silvie O., Greco C., Franetich J.F., Dubart-Kupperschmitt A., Hannoun L., van Gemert G.J., Sauerwein R.W., Levy S., Boucheix C., Rubinstein E., Mazier D. (2006). Expression of human CD81 differently affects host cell susceptibility to malaria sporozoites depending on the Plasmodium species. Cell. Microbiol..

[39] Risco-Castillo V., Topçu S., Son O., Briquet S., Manzoni G., Silvie O. (2014). CD81 is required for rhoptry discharge during host cell invasion by Plasmodium yoelii sporozoites. Cell. Microbiol..

[40] Volz J.C., Yap A., Sisquella X., Thompson J.K., Lim N.T.Y., Whitehead L.W., Chen L., Lampe M., Tham W.-H., Wilson D. (2016). Essential Role of the PfRh5/PfRipr/CyRPA Complex during Plasmodium falciparum Invasion of Erythrocytes. Cell Host Microbe.

[41] Wong W., Huang R., Menant S., Hong C., Sandow J.J., Birkinshaw R.W., Healer J., Hodder A.N., Kanjee U., Tonkin C.J. (2019). Structure of Plasmodium falciparum Rh5–CyRPA–Ripr invasion complex. Nature.

[42] Chen L., Xu Y., Wong W., Thompson J.K., Healer J., Goddard-Borger E.D., Lawrence M.C., Cowman A.F. (2017). Structural basis for inhibition of erythrocyte invasion by antibodies to plasmodium falciparum protein CyRPA. Elife.

[43] Reddy K.S., Amlabu E., Pandey A.K., Mitra P., Chauhan V.S., Gaur D. (2015). Multiprotein complex between the GPI-anchored CyRPA with PfRH5 and PfRipr is crucial for Plasmodium falciparum erythrocyte invasion. Proc. Natl. Acad. Sci. USA.

[44] Galaway F., Yu R., Constantinou A., Prugnolle F., Wright G.J. (2019). Resurrection of the ancestral RH5 invasion ligand provides a molecular explanation for the origin of P. Falciparum malaria in humans. PLoS Biol..

[45] Dreyer A.M., Beauchamp J., Matile H., Pluschke G. (2010). An efficient system to generate monoclonal antibodies against membrane-associated proteins by immunisation with antigen-expressing mammalian cells. BMC Biotechnol..

[46] Fernandes P., Loubens M., Le Borgne R., Marinach C., Ardin B., Briquet S., Vincensini L., Hamada S., Hoareau-Coudert B., Verbavatz J.-M. (2022). The AMA1-RON complex drives Plasmodium sporozoite invasion in the mosquito and mammalian hosts. PLoS Pathog..

[47] Kolli S.K., Salman A.M., Ramesar J., Chevalley-Maurel S., Kroeze H., Geurten F.G.A., Miyazaki S., Mukhopadhyay E., Marin-Mogollon C., Franke-Fayard B. (2021). Screening of viral-vectored P. falciparum pre-erythrocytic candidate vaccine antigens using chimeric rodent parasites. PLoS One.

[48] Jumper J., Evans R., Pritzel A., Green T., Figurnov M., Ronneberger O., Tunyasuvunakool K., Bates R., Žídek A., Potapenko A. (2021). Highly accurate protein structure prediction with AlphaFold. Nature.

[49] Silvie O., Charrin S., Billard M., Franetich J.F., Clark K.L., van Gemert G.J., Sauerwein R.W., Dautry F., Boucheix C., Mazier D., Rubinstein E. (2006). Cholesterol contributes to the organization of tetraspanin-enriched microdomains and to CD81-dependent infection by malaria sporozoites. J. Cell Sci..

[50] Klug D., Goellner S., Kehrer J., Sattler J., Strauss L., Singer M., Lu C., Springer T.A., Frischknecht F. (2020). Evolutionarily distant I domains can functionally replace the essential ligandbinding domain of plasmodium trap. Elife.

[51] Silvie O., Goetz K., Matuschewski K. (2008). A sporozoite asparagine-rich protein controls initiation of Plasmodium liver stage development. PLoS Pathog..

[52] Iwanaga S., Khan S.M., Kaneko I., Christodoulou Z., Newbold C., Yuda M., Janse C.J., Waters A.P. (2010). Functional Identification of the Plasmodium Centromere and Generation of a Plasmodium Artificial Chromosome. Cell Host Microbe.

[53] Schneider C.A., Rasband W.S., Eliceiri K.W. (2012). NIH Image to ImageJ: 25 years of image analysis. Nat. Methods.

[54] Buchan D.W.A., Jones D.T. (2019). The PSIPRED Protein Analysis Workbench: 20 years on. Nucleic Acids Res..

[55] Mulder N.J., Apweiler R., Attwood T.K., Bairoch A., Bateman A., Binns D., Biswas M., Bradley P., Bork P., Bucher P. (2002). InterPro: an integrated documentation resource for protein families, domains and functional sites. Brief. Bioinform..

[56] Söding J., Biegert A., Lupas A.N. (2005). The HHpred interactive server for protein homology detection and structure prediction. Nucleic Acids Res..

[57] Gíslason M.H., Nielsen H., Almagro Armenteros J.J., Johansen A.R. (2021). Prediction of GPI-anchored proteins with pointer neural networks. Curr. Res. Biotechnol..

[58] Mészáros B., Erdös G., Dosztányi Z. (2018). IUPred2A: Context-dependent prediction of protein disorder as a function of redox state and protein binding. Nucleic Acids Res..

[59] Heo L., Park H., Seok C. (2013). GalaxyRefine: Protein structure refinement driven by side-chain repacking. Nucleic Acids Res..

[60] Krieger E., Joo K., Lee J., Lee J., Raman S., Thompson J., Tyka M., Baker D., Karplus K. (2009). Improving physical realism, stereochemistry, and side-chain accuracy in homology modeling: Four approaches that performed well in CASP8. Proteins.

[61] Chen V.B., Arendall W.B., Headd J.J., Keedy D.A., Immormino R.M., Kapral G.J., Murray L.W., Richardson J.S., Richardson D.C. (2010). MolProbity: All-atom structure validation for macromolecular crystallography. Acta Crystallogr. D Biol. Crystallogr..

[62] Wiederstein M., Sippl M.J. (2007). ProSA-web: Interactive web service for the recognition of errors in three-dimensional structures of proteins. Nucleic Acids Res..

[63] Pettersen E.F., Goddard T.D., Huang C.C., Couch G.S., Greenblatt D.M., Meng E.C., Ferrin T.E. (2004). UCSF Chimera - A visualization system for exploratory research and analysis. J. Comput. Chem..

[64] Baker N.A., Sept D., Joseph S., Holst M.J., McCammon J.A. (2001). Electrostatics of nanosystems: Application to microtubules and the ribosome. Proc. Natl. Acad. Sci. USA..

[65] Dolinsky T.J., Nielsen J.E., McCammon J.A., Baker N.A. (2004). PDB2PQR: An automated pipeline for the setup of Poisson-Boltzmann electrostatics calculations. Nucleic Acids Res..

[66] Katoh K., Standley D.M. (2013). MAFFT Multiple Sequence Alignment Software Version 7: Improvements in Performance and Usability. Mol. Biol. Evol..

[67] Guindon S., Dufayard J.-F., Lefort V., Anisimova M., Hordijk W., Gascuel O. (2010). New Algorithms and Methods to Estimate Maximum-Likelihood Phylogenies: Assessing the Performance of PhyML 3.0. Syst. Biol..

[68] Lefort V., Longueville J.-E., Gascuel O. (2017). SMS: Smart Model Selection in PhyML. Mol. Biol. Evol..

[69] Anisimova M., Bielawski J.P., Yang Z. (2001). Accuracy and power of the likelihood ratio test in detecting adaptive molecular evolution. Mol. Biol. Evol..

[70] Huang Y.F., Golding G.B. (2014). Phylogenetic Gaussian Process Model for the Inference of Functionally Important Regions in Protein Tertiary Structures. PLoS Comput. Biol..

[71] Langella O., Valot B., Balliau T., Blein-Nicolas M., Bonhomme L., Zivy M. (2017). X!TandemPipeline: A Tool to Manage Sequence Redundancy for Protein Inference and Phosphosite Identification. J. Proteome Res..

[72] Ramakrishnan C., Delves M.J., Lal K., Blagborough A.M., Butcher G., Baker K.W., Sinden R.E. (2013). Laboratory maintenance of rodent malaria parasites. Methods Mol. Biol..

[73] Janse C.J., Ramesar J., Waters A.P. (2006). High-efficiency transfection and drug selection of genetically transformed blood stages of the rodent malaria parasite Plasmodium berghei. Nat. Protoc..

[74] Prudêncio M., Rodrigues C.D., Ataíde R., Mota M.M. (2008). Dissecting in vitro host cell infection by Plasmodium sporozoites using flow cytometry. Cell. Microbiol..

[75] Narum D.L., Thomas A.W. (1994). Differential localization of full-length and processed forms of PF83/AMA-1 an apical membrane antigen of Plasmodium falciparum merozoites. Mol. Biochem. Parasitol..

[76] Schindelin J., Arganda-Carreras I., Frise E., Kaynig V., Longair M., Pietzsch T., Preibisch S., Rueden C., Saalfeld S., Schmid B. (2012). Fiji: An open-source platform for biological-image analysis. Nat. Methods.

[77] Brown K.M., Sibley L.D., Lourido S. (2020). Methods in Molecular Biology.

[78] Perez-Riverol Y., Bai J., Bandla C., García-Seisdedos D., Hewapathirana S., Kamatchinathan S., Kundu D.J., Prakash A., Frericks-Zipper A., Eisenacher M. (2022). The PRIDE database resources in 2022: A hub for mass spectrometry-based proteomics evidences. Nucleic Acids Res..

[79] Callebaut I., Labesse G., Durand P., Poupon A., Canard L., Chomilier J., Henrissat B., Mornon J.P. (1997). Deciphering protein sequence information through hydrophobic cluster analysis (HCA): Current status and perspectives. Cell. Mol. Life Sci..

[80] Aurrecoechea C., Brestelli J., Brunk B.P., Dommer J., Fischer S., Gajria B., Gao X., Gingle A., Grant G., Harb O.S. (2009). PlasmoDB: A functional genomic database for malaria parasites. Nucleic Acids Res..

