# Supplementary material for: Plasmodium sporozoites require the protein B9 to invade hepatocytes
Source: iScience. 2023 Jan 25;26(2):106056. doi: 10.1016/j.isci.2023.106056 (PMC9906020; doi:10.1016/j.isci.2023.106056)
Supplement: Document S1. Figures S1–S11 [file mmc1.pdf]

iScience, Volume *26*

Supplemental information

Plasmodium sporozoites require the protein B9 to invade hepatocytes

Priyanka Fernandes, Manon Loubens, Carine Marinach, Romain Coppée, Ludivine Baron, Morgane Grand, Thanh-Phuc Andre, Soumia Hamada, Anne-Claire Langlois, Sylvie Briquet, Philippe Bun, and Olivier Silvie

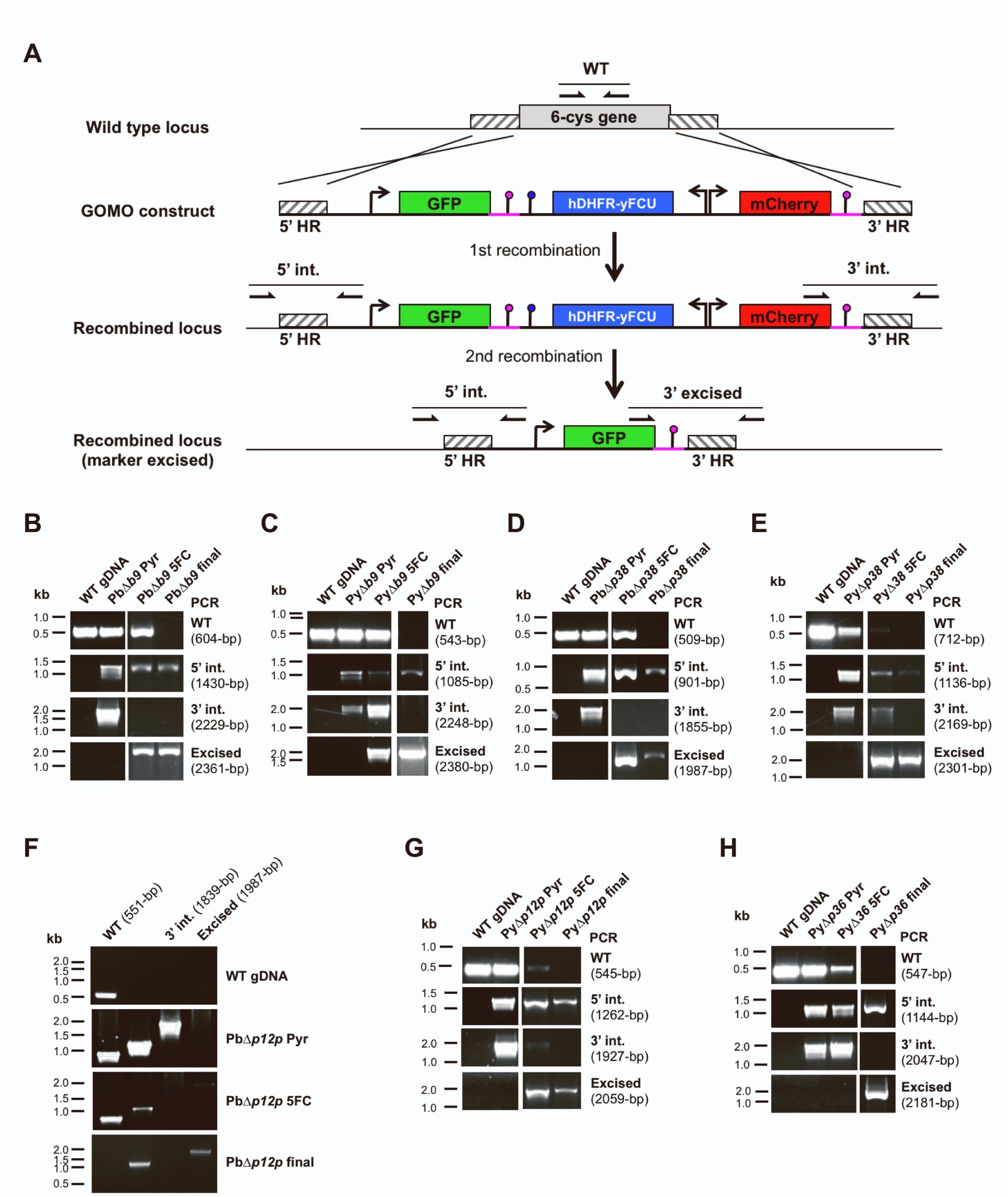

Figure S1. Generation of 6-cys knockout parasite lines in *P. berghei* and *P. yoelii*. Related to Figure 1. (A) Replacement strategy to delete 6-cys candidate genes. The wild-type locus of 6-cys genes was targeted with a GOMO-GFP replacement plasmid containing a 5' and a 3' homologous sequence inserted on each side of a GFP/hDHFR-yFCU/mCherry triple cassette. Upon double crossover recombination, the gene of interest is replaced by the plasmid cassettes. Subsequent recombination between the two identical PbDHFR/TS 3' UTR sequences (pink lollipops) results in excision of hDHFR-yFCU and mCherry. Genotyping primers and expected PCR fragments are indicated by arrows and lines, respectively. (B–H) Genotyping of WT and PbΔ*b9* (B), PyΔ*b9* (C), PbΔ*p38* (D), PyΔ*p38* (E), PbΔ*p12p* (F), PbΔ*p12p* (G) and PbΔ*p36* (H) parasites, recovered after positive selection with pyrimethamine (Pyr), negative selection with 5-fluorocytosine (5FC), and parasite sorting by flow cytometry (final).

Parasite genomic DNA was analyzed by PCR using primer combinations specific for the unmodified locus (WT), the 5' integration (5'int.), 3' integration (3'int.) and 3' marker excision (excised) events. The absence of amplification with primer combinations specific for the WT locus (WT) and the non-excised integrated construct (3' integration) confirms that the final populations contain pure knockout drug-selectable marker-free parasites.

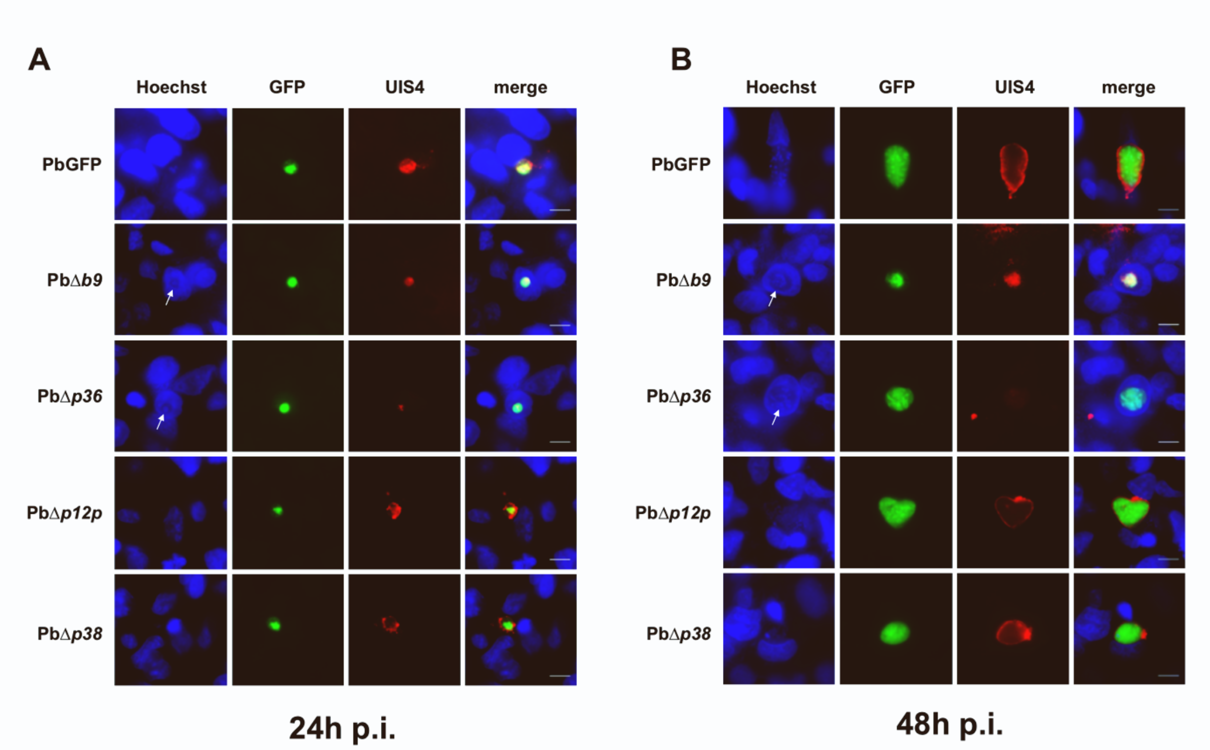

Figure S2. Development of 6-cys *P. berghei* knockout parasite lines *in vitro.* Related to Figure 1. (A and B) Immunofluorescence images of HepG2 cells infected with PbGFP, PbΔ*b9*, PbΔ*p36*, PbΔ*p12p* or PbΔ*p38* parasites expressing GFP (green) and labelled with anti-UIS4 antibodies (red) and Hoechst 33342 (blue), 24 h (A) or 48 h (B) post-infection. PbGFP, PbΔ*p12p* and PbΔ*p38* are surrounded by a UIS4-positive PV membrane (red), while PbΔ*p36* and PbΔ*b9* parasites are localized inside the nucleus of the infected cell (arrows) and lack a UIS4-positive PVM. Scale bars, 10 µm.

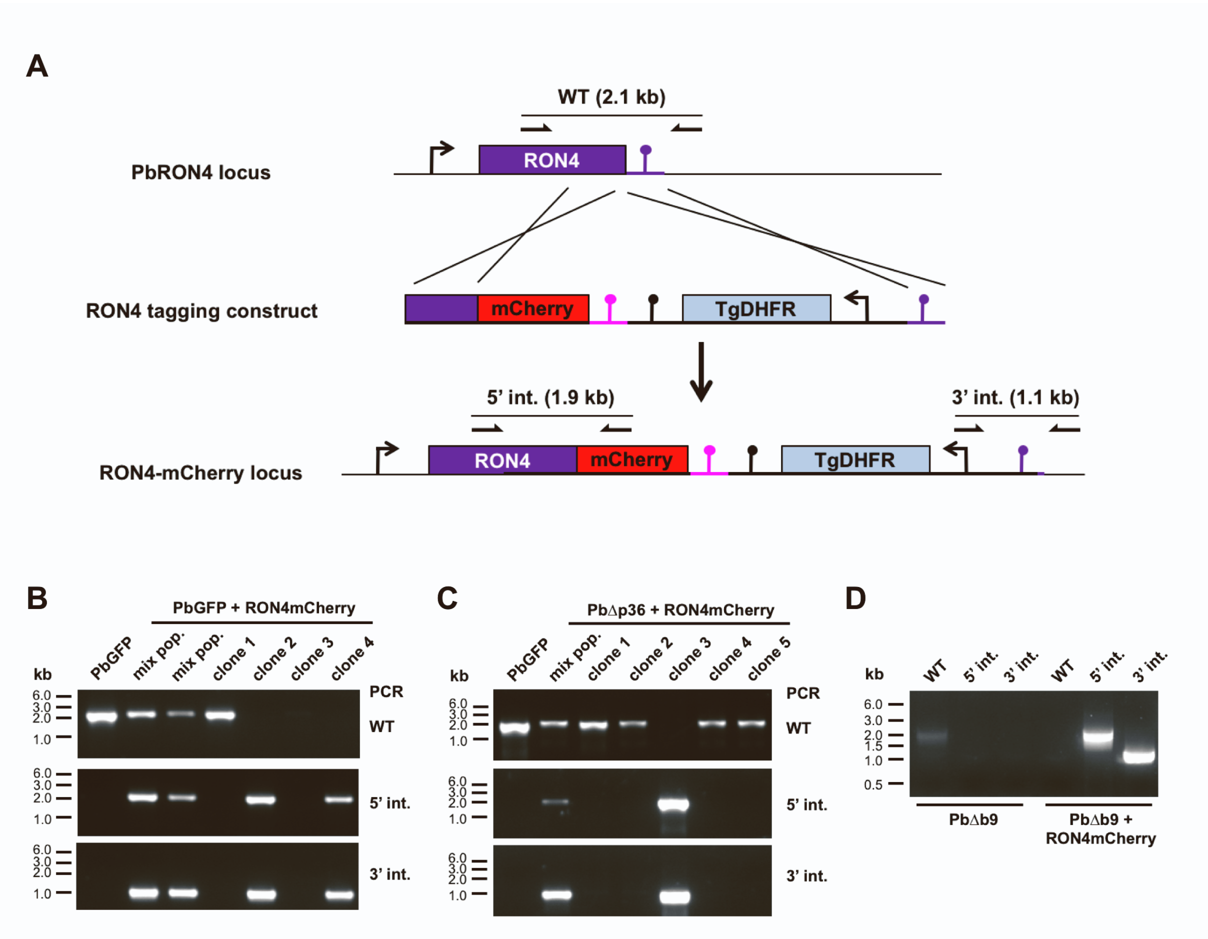

Figure S3. Generation of RON4-mCherry expressing parasites. Related to Figure 2. (A) Strategy used to tag RON4 with mCherry by double crosser homologous recombination in PbGFP, PbΔ*p36* and PbΔ*b9* parasites. The *P. berghei RON4* locus was targeted with a tagging construct containing a 5' homology fragment coding the C-terminal part of RON4, fused in frame to the mCherry coding sequence and followed by the 3' UTR of *P. berghei* DHFR (pink lollipop), a TgDHFR/TS selection cassette, and a 3' homology fragment corresponding to *RON4* 3'UTR (purple lollipop). Upon a double crossover event, the endogenous *RON4* gene is replaced by a single mCherry-tagged *RON4* copy. Genotyping primers and expected PCR fragments are indicated by arrows and lines, respectively. (B-D) Correct construct integration was confirmed by analytical PCR using primers specific for the unmodified locus (WT) or for the 5' and 3' recombination events (5' int. and 3' int., respectively) at the *RON4* locus. The absence of amplification with the WT primer combination confirms the purity of the transgenic population in PbGFP/RON4-mCherry clones 2 and 4 (B), PbΔ*p36*/RON4-mCherry clone 3 (C) and PbΔ*b9*/RON4-mCherry (D).

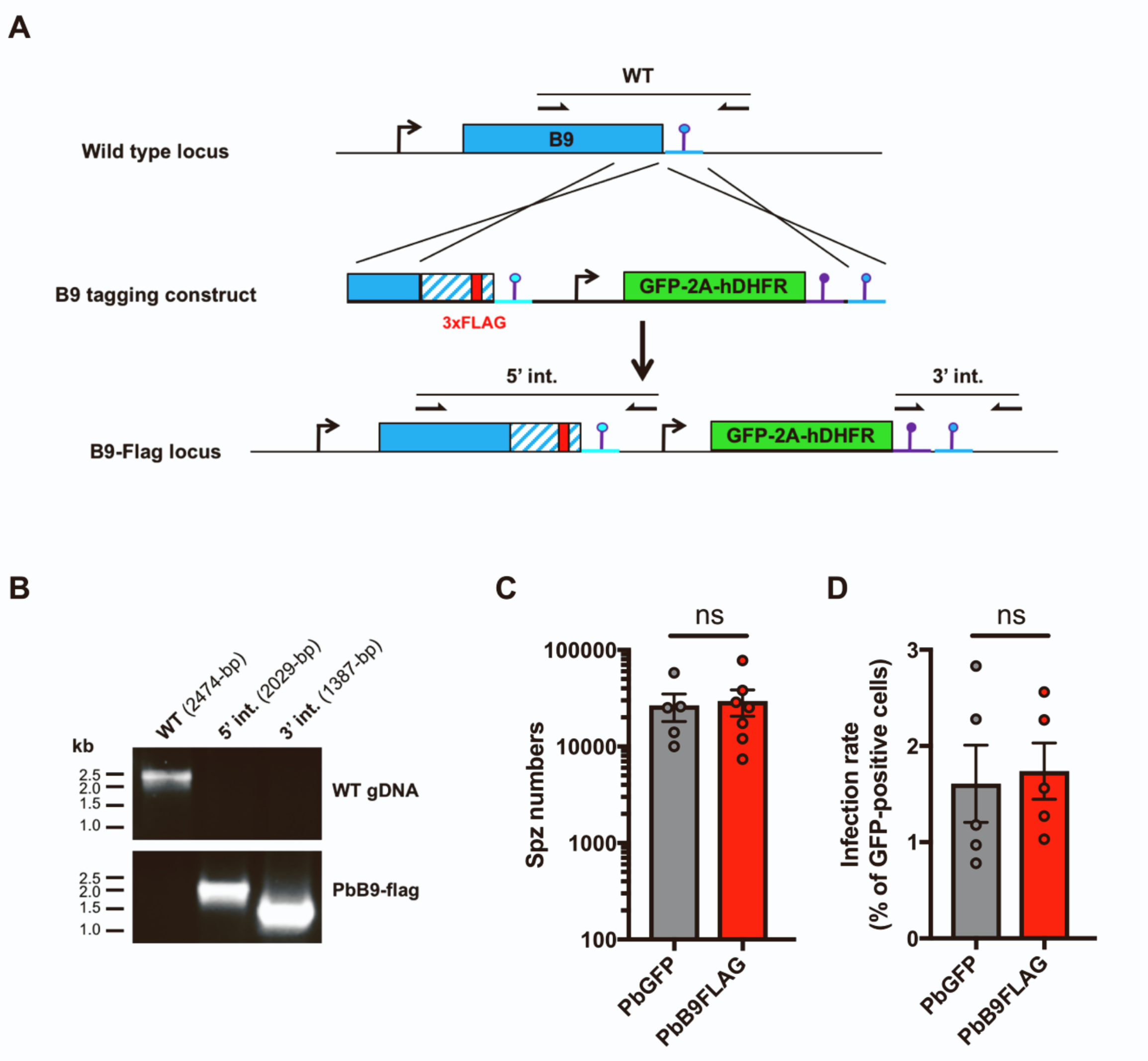

Figure S4. Genetic tagging of B9 in *P. berghei*. Related to Figure 3. (A) Strategy used to tag B9 with a triple Flag epitope by double crosser homologous recombination in *P. berghei* WT parasites. The *P. berghei B9* locus was targeted with a tagging construct containing a 5' homology fragment from PbB9 ORF, a recodonized C-terminal sequence of B9 (blue and white striped) with a 3xFlag sequence inserted (red), the 3' UTR of *PyB9* (cyan lollipop), a GFP-2A-hDHFR cassette, and a 3' homology fragment corresponding to *PbB9* 3'UTR (blue lollipop). Upon a double crossover event, the endogenous *B9* gene is replaced by a single Flag-tagged *B9* copy. Genotyping primers and expected PCR fragments are indicated by arrows and lines, respectively. (B) Correct construct integration was confirmed by analytical PCR using primers specific for the unmodified locus (WT) or for the 5' and 3' recombination events (5' int. and 3' int., respectively) at the *B9* locus. The absence of amplification with the WT primer combination confirms the purity of the transgenic population in PbB9-Flag parasites. (C) Number of sporozoites isolated from the salivary glands of mosquitoes infected with PbGFP or PbB9-Flag parasites (mean +/- SEM; $p = 0.2893$, two-tailed ratio paired t test). (D) Infection rates of PbGFP and PbB9-Flag parasites were determined in HepG2 cells 24 hours post-infection. The results show the percentage of invaded (GFP-positive) cells as determined by FACS (mean +/- SEM; $p = 0.6768$, two-tailed ratio paired t test).

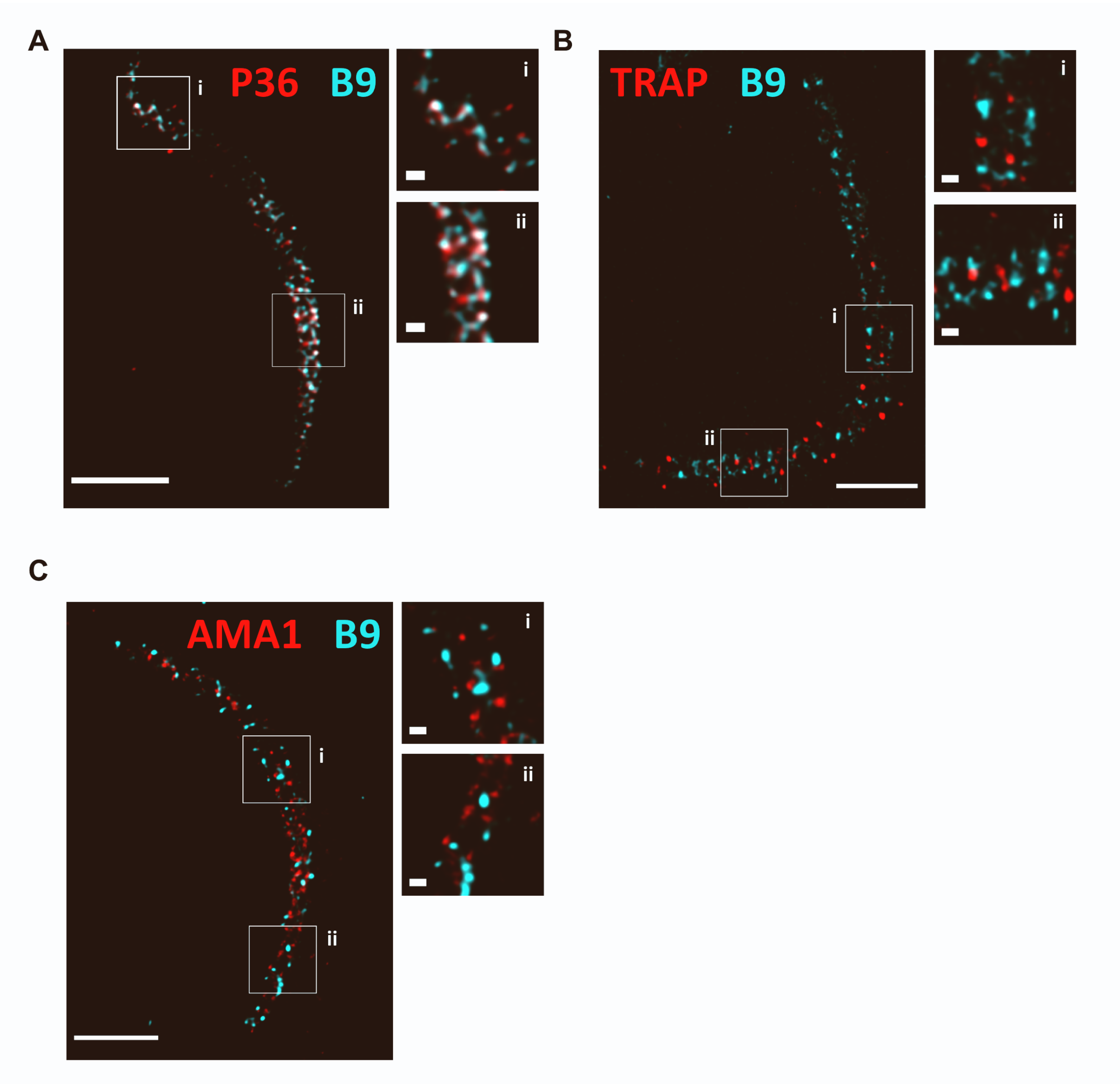

Figure S5. B9 colocalizes with P36 but not with TRAP or AMA1 in *P. berghei* sporozoites. Related to Figure 3. (A) STED images of sporozoites expressing B9-Flag and P36-mCherry, labelled with anti-mCherry (red) and anti-Flag (cyan) antibodies. (B and C) STED images of B9-Flag sporozoites labelled with anti-Flag (cyan) and anti-TRAP (B, red) or anti-AMA1 (C, red) antibodies. Scale bars, 2 µm (200 nm in insets).

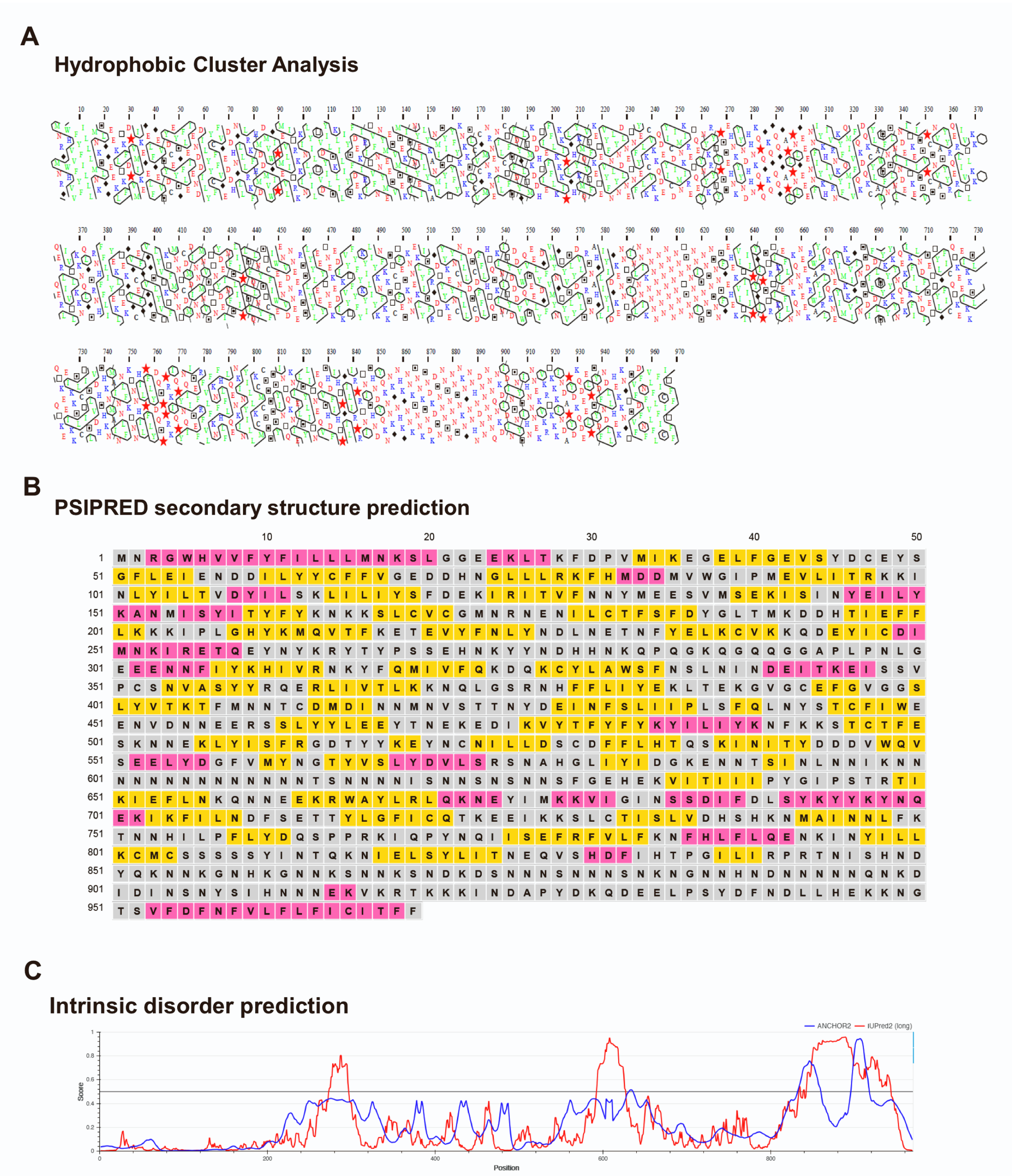

Figure S6. Secondary structure analysis of PfB9. Related to Figure 4. (A) Hydrophobic cluster analysis of PfB9. Cluster of hydrophobic amino acids are surrounded. Red stars and black diamond correspond to proline and glycine amino acids respectively. (B) Secondary structure prediction PfB9 using PSIPRED 4.0. Pink, yellow, and grey background colors indicate predicted helix, strand, and coil structures, respectively. (C) Intrinsic disorder prediction of PfB9 using IUPred2A. Predictions are based on energy estimation for ordered and disordered residues by IUPred2 (red line) and for disordered binding regions by ANCHOR2 (blue line).

A

Molprobity results

	PfB9 propeller model	CyRPA
**Poor rotamers**	1/341 (0.29)	2/305 (0.66)
**Favored rotamers**	335/341 (97.95)	283/305 (92.79)
**Ramachandran outliers**	4/359 (1.11)	0/320 (0.00)
**Ramachandran favored**	330/359 (91.92)	307/320 (95.94)
**Bad bonds**	5/3121 (0.16)	0/2775 (0.00)
**Bad angles**	6/4204 (0.14)	0/3744 (0.00)

B

Prosa II result

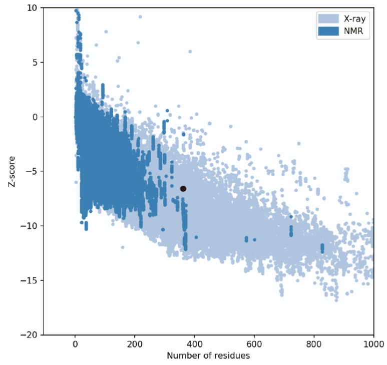

Figure S7. Model structure validation. Related to Figure 4. The structure model of PfB9 propeller (amino acids 26 to 386) was validated using MolProbity (A) and Prosa II (B). For Prosa II, the Z-score of the PfB9 propeller model (indicated as a black point) falls within those of protein structures obtained by X-ray crystallography.

```
      *~**~*~~~~**~*~~~*~*~*.***~~.~**.~**~******..~.~-***..~~*~~~~~~*~..~***~~~~~~*..*~~~~*.*.~~*****~~~~~~~~******~*~*~~*~**~.**
PfB9  LTKFDPVMIKEGELFGEVSYDCEYSGFLEIENDDILYYCFFVGEDDHNGLLLRKFHMDDMVWGIPMEVLITRK-KINLYILTVDYILSKLILIYSFDEKIRITVFNNYMEESVMSEKISINY
PbB9  LNKFNPIIKEEGYLYLKVAYNCEYSGLIGLENNYILHYCFFVSNAT-NGLSLTTFNTLKLKWDIPKEVLLFKNDNIQGYSFITDFISNNLILIYDINNITKVTVFNNYIEPSIISNKITVNY
PyB9  LNKFNPIIKEEGYLYLKVAYNCDYSGLIELENIYILHYCFFINNTT-NGLLFTTFNTLKLKWDVAKEVLLFKNDNIERYSFITDSIANNLILIYDINNITKVTVFNNYIEPSIISNKITTNY

      ~~.~*~*~**~~**********~~****~*~**.*~*********.*~~~.**~**~~**~~~**~~*~*.~~~*****~~~~.*~~***~**~~~~~~~*~**~~*~*~~~~~--------
PfB9  EILYKANMISYITYFYKNKKSLCVCGMNRNENILCTFSFDYGLTMKDDHTIEFFLKKKIPLGHYKMQVTFKETEVYFNLYNDLNETNFYELKCVKK-QDEYICDIMNKIRETQEYNYKRYTY
PbB9  KMVHKFNIISKVTYFYKNKKSLFMCGMNNNGNIICSFSFDYGLTMTDENLVEFILKDSIPIIQYKIDVKFKKHYVYFNLREEKKEISFYEFKCFQDTENSYTCDLLNIISANLQ--------
PyB9  KMIHKFNIISKVTYFYKNKKSLFMCGMNNNGNIVCSFSFDYGLTMKDENLIEFILKDSIPIIQYKIDVKFNKHYVYFNLREEKNEISFYEFKCFQDTENTYTCDLLNIISANLQ--------

      -------------------------------------*~.**~*~*~*~~*~~~~**~~~**~~*********~**~*~**~~.*~**~~.~..*.*~.*~***~~~~~.~~.~~~~~~***
PfB9  PSSEHNKYYNDHHNKQPQGKQGQQGGAPLPNLGEEENNFIYKHIVRNKYFQMIVFQKDQKCYLAWSFNSLNINDEITKEISSVPCSNVASYYRQERLIVTLKKN---QLGSRNHFFLIYEKL
PbB9  -------------------------------------NIKYKYILRTKDSQIVSYQKGNICYIGWSFNSLNINTEIDKQISDTECFNVSIVQMNENLLATFKKNINPDDKTGNEYYALFEKL
PyB9  -------------------------------------NINYKYILRTKDSQIVSYQKGNICYIGWSFNSLNINTEIDKQISDTDCFNVSILQKDEHLLVTFKKNINPDDPTGKEYYALFEKL
```

```
*  Full identity between PfB9, PbB9 and PyB9
~  Identity between PbB9 and PyB9
.  Mismatch between PbB9 and PyB9
-  Gapped positions of PbB9 and PyB9
```

Figure S8. Protein sequence alignment of the B9 propeller from *P. falciparum*, *P. berghei* and *P. yoelii*. Related to Figure 4. Conserved residues are in bold, cysteines in red.

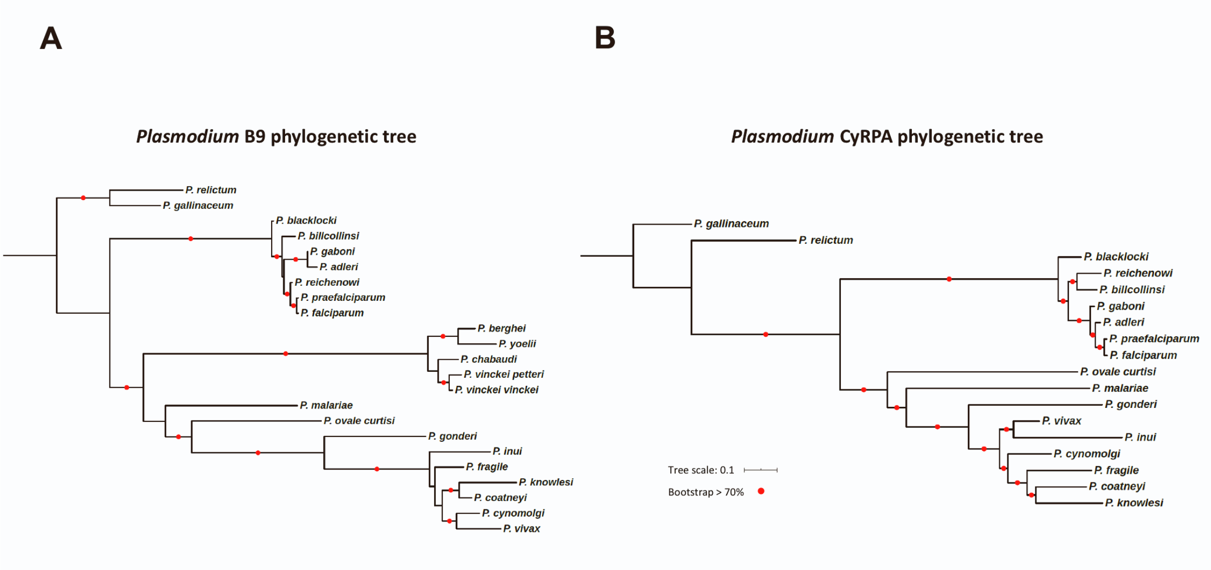

Figure S9. Phylogenetic trees of B9 and CyRPA. Related to Figure 4. Multiple sequence alignments and corresponding phylogenetic trees were established using two datasets consisting of distinct *Plasmodium* B9 (A, n = 23) or CyRPA (B, n = 18) sequences. Phylogenetic trees were inferred by maximum likelihood using PhyML, and branch supports were estimated using the approximate likelihood ratio aLRT SH-like method.

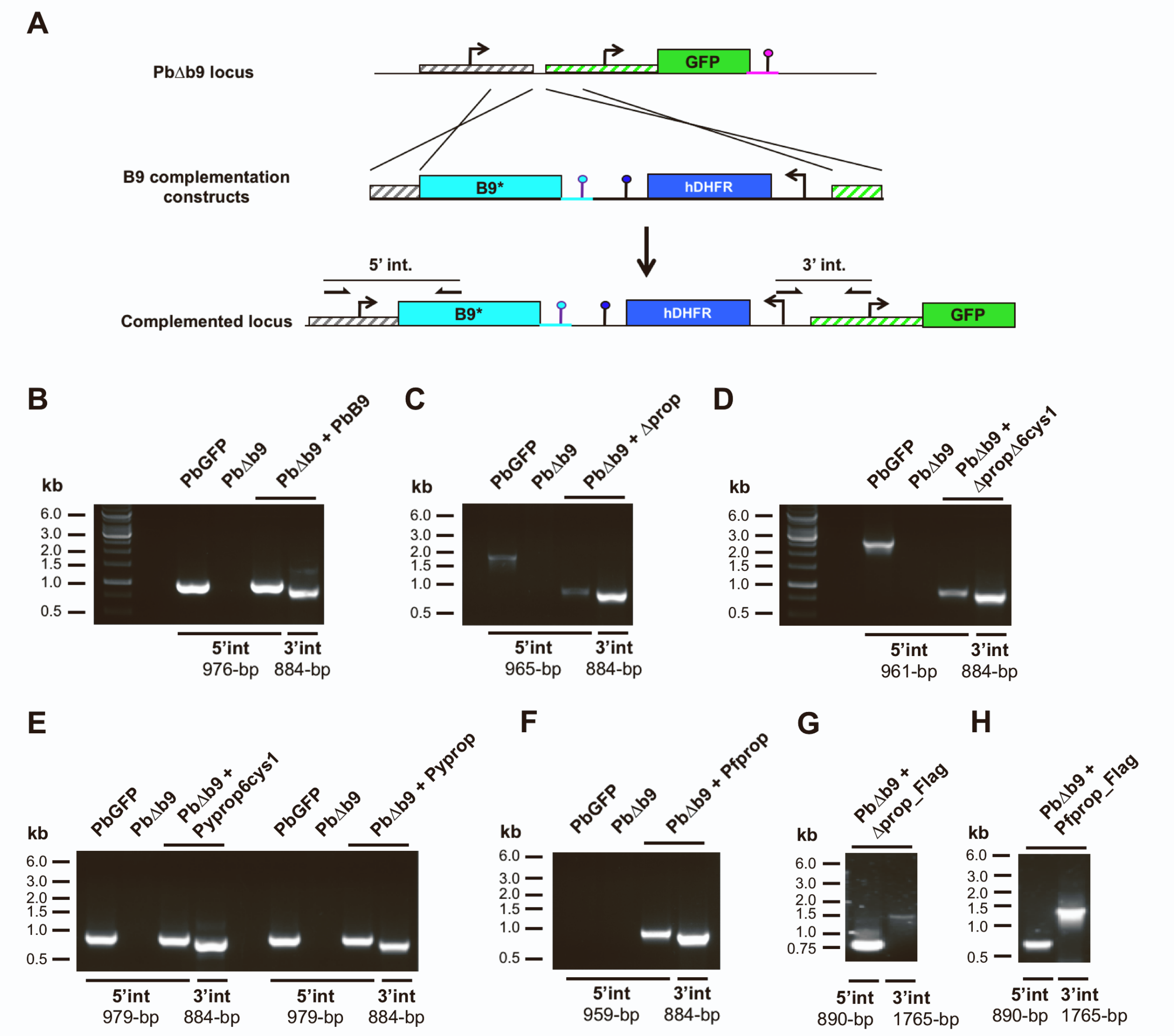

Figure S10. Genetic complementation of PbΔb9 parasites. Related to Figure 5. (A) Strategy used to genetically complement PbΔ*b9* with different versions of B9 (indicated as B9*) by double crossover homologous recombination. Genotyping primers and expected PCR fragments are indicated by arrows and lines, respectively, but are not at scale. (B-H) Correct construct integration was confirmed by analytical PCR using primers specific for the 5' and 3' recombination events (5' int. and 3' int., respectively) and genomic DNA from PbGFP, PbΔ*b9*, and PbΔ*b9* complemented with the PbB9 (B), Δprop (C), ΔpropΔ6cys (D), Pyprop (E), Pyprop6cys1 (E), Pfprop (F), Δprop-Flag (G) or Pfprop-Flag (H) constructs.

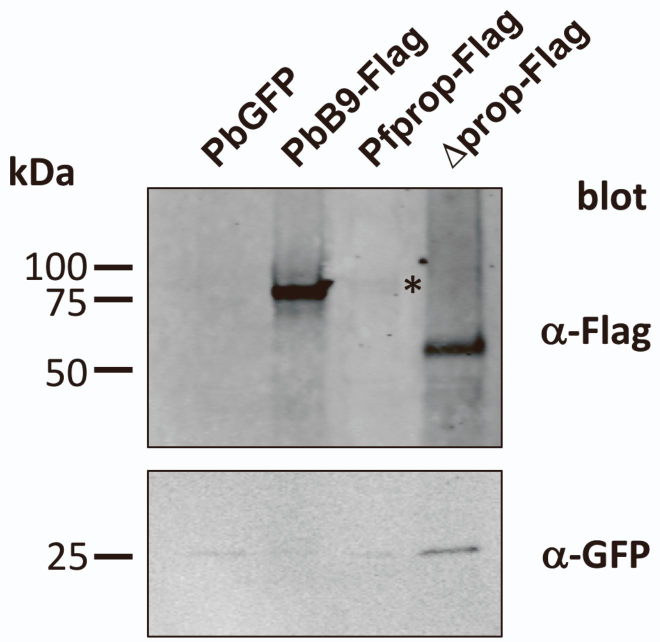

Figure S11. Western blot analysis of Δprop-Flag and Pfprop-Flag protein expression. Related to Figure 5. Immunoblot of sporozoite lysates from PbGFP (control), PbB9-Flag and PbΔ*b9* parasites complemented with Pfprop-Flag or Δprop-Flag constructs, using anti-Flag or anti-GFP antibodies. The anti-Flag blot shows the presence of bands for PbB9-Flag (theoretical size ~95 kDa), Pfprop-Flag (theoretical size ~100 kDa), and Δprop-Flag (theoretical size ~58 kDa), which are absent in the PbGFP control lane, confirming the specificity of the labelling. The PfProp-Flag protein was detected as a faint band, indicated by an asterisk. Relative quantification using the GFP band for normalization revealed lower expression of Pfprop-Flag (5%) and Δprop-Flag (7%) as compared to PbB9-Flag (100%).
